# A global yield dataset for major lignocellulosic bioenergy crops based on field measurements

**DOI:** 10.1038/sdata.2018.169

**Published:** 2018-08-21

**Authors:** Wei Li, Philippe Ciais, David Makowski, Shushi Peng

**Affiliations:** 1 Laboratoire des Sciences du Climat et de l’Environnement, LSCE/IPSL, CEA-CNRS-UVSQ, Université Paris-Saclay, Gif-sur-Yvette 91191, France; 2 UMR Agronomie, INRA, AgroParisTech, Université Paris-Saclay, Thiverval-Grignon 78850, France; 3 Sino-French Institute for Earth System Science, College of Urban and Environmental Sciences, Peking University, Beijing 100871, China

**Keywords:** Energy economics, Carbon cycle, Agroecology

## Abstract

Reliable data on biomass produced by lignocellulosic bioenergy crops are essential to identify sustainable bioenergy sources. Field studies have been performed for decades on bioenergy crops, but only a small proportion of the available data is used to explore future land use scenarios including bioenergy crops. A global dataset of biomass production for key lignocellulosic bioenergy crops is thus needed to disentangle the factors impacting biomass production in different regions. Such dataset will be also useful to develop and assess bioenergy crop modelling in integrated assessment socio-economic models and global vegetation models. Here, we compiled and described a global biomass yield dataset based on field measurements. We extracted 5,088 entries of data from 257 published studies for five main lingocellulosic bioenergy crops: eucalypt, *Miscanthus*, poplar, switchgrass, and willow. Data are from 355 geographic sites in 31 countries around the world. We also documented the species, plantation practices, climate conditions, soil property, and managements. Our dataset can be used to identify productive bioenergy species over a large range of environments.

## Background & Summary

Bioenergy crops provide renewable energy and play a vital role for future energy sustainability^1^. Bioenergy is also a pivotal option of climate change mitigation solutions as biomass can be substituted for the use of fossil fuels^2^. Bioenergy combined with carbon capture and storage (BECCS), a technology that is not fully implemented today, is often seen as an indispensable component of negative emission technology in Integrated Assessment Models (IAMs) to develop low climate warming emission scenarios^2–5^. Most recent studies on bioenergy rely on energy sources derived from lignocellulosic crops that include productive perennial grasses (e.g. Miscanthus and switchgrass) and fast-growing trees (e.g. eucalypt, poplar and willow), rather than on first generation biofuels based on grain or high-sugar crops^6^. Lignocellulosic crops can grow in a broad range of climate conditions which avoids direct competition for fertile lands with food crops^1^. There is also evidence that some crops like Miscanthus have low requirements for nutrient and fertilizer application^7,8^, and are characterized by low N2O emissions. Although reviews and meta-analysis were already conducted for several bioenergy crops^6–14^, there are still many challenges in understanding the factors impacting bioenergy crop yields at global scale owing largely to lack of global observation-based dataset of biomass yields with corresponding information on species, genotypes, climate conditions, soil properties and management practices.

In global vegetation models^15–17^, a proper representation of dedicated bioenergy crops is a prerequisite for accurately simulating the future dynamics of land carbon since bioenergy plantation has been increasingly deployed in future scenarios by IAMs^18–20^. A global bioenergy crop yield dataset based on field measurements would be valuable to assess performance of vegetation models, improve their structure, and calibrate their parameters. Such global dataset can be also used for providing observation-based bioenergy crop yields to IAMs after proper statistical analysis and upscaling.

**Figure 1:**
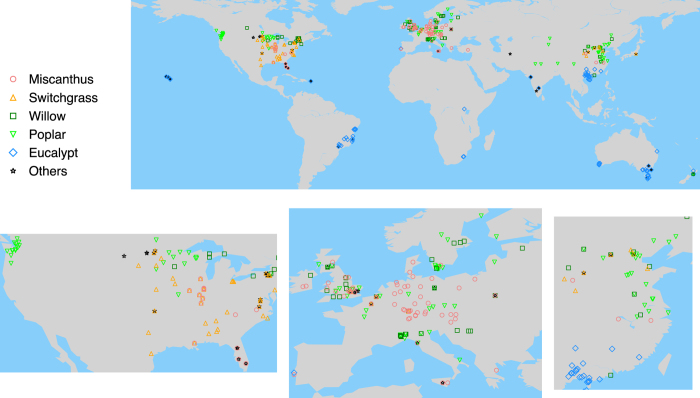
Locations of the yield data. The lower panels show the zoom-in maps of North America, Europe and East Asia, respectively.

Field trials and field measurements of lignocellulosic bioenergy crops have been conducted around the world during the past decades. A dataset including results extracted from 28 papers for 36 bioenergy crop species was already published^10^. However, this dataset was built to compare species on similar environments, and includes results of trials comparing at least two different species^10^. Because of this constraint, this dataset covered a limited number of experimental sites located in 12 countries^10^. Here, we constructed a global bioenergy biomass yield dataset based on field measurements for five major bioenergy crop types: eucalypt, *Miscanthus*, poplar, switchgrass, and willow. These five crops represent the most promising and economically important herbaceous and woody bioenergy crops^1,6,11,15^. They have been studied in numerous field experiments. We extracted bioenergy crop yield data from 257 published studies. The total number of geographic locations is 355 (Fig. 1) covering 31 countries over different regions around the world (Table 1). The spatial distribution of data for different plant types (Fig. 1) reflects the availability of studies in each region, related to suitable plant traits for local climate conditions and possibly to policy and economic rationale fostering research on different crop types in different regions. For example, very few studies are available in Africa partly because of the limited research activity on bioenergy crops in this continent, and most of the eucalypt data were collected in tropical and sub-tropical regions where this crop type is commonly grown (Fig. 1,Table 1). There are 5,088 entries in our dataset, each entry being one reported biomass yield value in mass unit (ton DM/yr, dry matter). Each entry represents the biomass yield, and each yield data is characterized by attributes such as site location, climate, soil property, plantation (e.g. planting date, harvest date, age, rotation length, planting density), and management techniques (irrigation and different fertilizer applications) (see details in Data Records).

This dataset is freely available. It can be used in future meta-analyses to understand factors impacting bioenergy crop yields and identifying the most productive species. The dataset can also be used for assessing and improving IAMs and global vegetation models. In addition, our yield data can be potentially upscaled to a global observation-based bioenergy yield map that would provide valuable information for scientists in agriculture and climate change and for policy makers to determine future land use planning.

## Methods

### Literature search and data extraction

We conducted a systematic literature search of studies on observation-based biomass yields for bioenergy crops. We targeted five main bioenergy crop types: eucalypt (*Eucalyptus spp.*), poplar (*Populus spp.*), willow (*Salix spp.*), *Miscanthus* (*Miscanthus spp.*) and switchgrass (mainly *Panicum virgatum*).

We searched in Web of Science (https://www.webofknowledge.com/) in December 2016 using the keywords included in the following search equation: (“bioenergy crop” OR “biofuel crop” OR “eucalypt” OR “eucalyptus” OR “poplar” OR “populus” OR “willow” OR “Salix” OR “Miscanthus” OR “switchgrass” OR “Panicum”) AND (“biomass” OR “yield” OR “production”) AND (“field” OR “measurement” OR “trial” OR “observation”). No other restriction was considered on the publication date, language and other filtering criteria. This gives a total of 3,028 articles. We further went through the titles and abstracts of these articles and screened out those not fitting our criteria: 1) no mention of crop types, or different plant types from our selection; 2) data not from field or experiment studies (e.g. yields simulated by models); 3) no measured biomass or yield. We also carried out supplementary search for the review or meta-analysis studies on different research aspects of bioenergy crops and downloaded about 60 such studies. If the references cited in these reviews and meta-analyses fit our criteria but were not identified by our initial search, they were further selected. After literature searching and initial selections, we downloaded 459 eligible full-text articles.

In addition, we searched the China Knowledge Resource Integrated Database (http://www.cnki.net/) using the same search equations but in Chinese. Likewise, we downloaded 123 articles after an initial selection of titles and abstracts. Most of these articles are written in Chinese but with titles and abstracts in English.

Full texts of the 582 downloaded articles were examined in details to find out those meeting the following criteria: 1) the coordinates of field sites must be reported or can be retrieved from Google Map using the reported location information; 2) the biomass or yields must be directly reported in mass density units (e.g. ton DM ha^-1^) either by weighting the collected biomass after drying or estimated using empirical equations from e.g. diameter at breast height (DBH), and thus yields reported only in volume density units (e.g. m^3^ ha^-1^) are excluded; 3) crop ages (for yield data) and time increments (for biomass increase data) should be reported; 4) data are not duplicated in other articles. Therefore, each entry must have site coordinates, crop type and yield, and other information if available.

The selected articles meeting all the criteria are 257 in total (Data Citation 1). We went through the full-text of each article and extracted all the information needed (Table 2): site location, climate, soil property, plantation (e.g. plantation density, rotation length, and crop type), yield, and management techniques (e.g. irrigation and fertilization). Although we mainly focused on eucalypt, poplar, willow, *Miscanthus* and switchgrass, we also extracted yields of other lignocellulosic bioenergy crops (e.g. reed and sudangrass) when they were reported in the same studies.

### Code availability

The dataset is saved in a generic .txt format as well as the .xlsx format (Data Citation 1) that is easy to access by Microsoft Excel or other data processing software like R (https://cran.r-project.org/) or Python (https://www.python.org/). There are 5,088 entries with information in 41 columns (Table 2) in the dataset. Each entry represents one biomass yield data. In addition, we also provided an evaluation report using Python to show the basic information and statistics of each column in the dataset (see codes and results in Supplementary File 1).

## Data Records

Data records are reported in a single table including 5,088 rows and 41 columns. Each row corresponds to a single yield data, and each column corresponds to one variable describing location, plantation, or management information. The names, units and description of the columns are shown in Table 2. Columns are grouped by categories further denoted as “attributes” (Table 2).

Attribute 1 “Reference” contains one column reporting author names and publication year. Some entries have more than one reference, usually published by the same research group. This is because we compiled a variety of information (see column names in Table 2) corresponding to different field characteristics, and such information may be reported in different articles. For example, authors may report yield data in one article while report site location, fertilization information in another article for the same field study. In this case, we combined information from both articles in this single row, and that’s why there are more than one reference for this entry.

Attribute 2 “Site Location” contains six columns; “Country”, “Site”, “Coordinate_origin”, “Latitude”, “Longitude” and “Elevation”.

– “Country” reports the country where the field sites were located.
– “Site” reports location name (e.g. state and county).
– “Coordinate_origin” indicates the origin of “Latitude” and “Longitude” (directly reported site, center of reported area or identified from Google Map using the reported location information).
– “Latitude” and “Longitude” are systematically reported in the selected papers (see **Methods** section). The articles either directly reported the coordinates of the site location or provided the range of latitudes and longitudes for the field. In the latter case, the medians of latitudes and longitudes, representing the field center, were recorded in “Latitude” and “Longitude” attributes, respectively.
– “Elevation” is the field site elevation reported in the articles.

Attribute 3 “Climate” of each field site includes

– “Temperature” (reported mean annual temperature, MAT)
– “Rainfall” (reported mean annual precipitation, MAP).

Attribute 4 “Soil” of each field site includes column “Clay” which is the clay fraction reported.

Attribute 5 “Plantation” contains eleven columns.

– “Field_type” documents the field types of the observations, including experimental trial, farmer’s field or natural field.
– “Field_size” records the size of each plot in the experimental trials, the sampling plot from large-scale field or the total area of the field depending on what was reported in the original articles.
– “Crop_type” refers to the general bioenergy crop types. The five main types are labeled as “Eucalyptus”, “Poplar”, “Willow”, “Miscanthus” and “Switchgrass”. The other crops are labeled in their common names respectively, e.g. “giant reed” and “Sudangrass”.
– “Species” records the unique species names of each bioenergy crop.
– “Detailed_species_information” records the species names with other detailed information like cultivars and genotypes. Level of details depends on information reported in the original articles.
– “Planting_date” is the year and month of plantation.
– “Harvest_year” and “Harvest_date” record the year and the date (usually only the month) of harvest or of yield observation.
– “Age” is the age (in years) of bioenergy crops when harvest or observations were conducted. It should be noted that “Age” is not necessary difference between plantation year and harvest year for perennial grasses, but could correspond to difference between plantation year and observation year.
– “Rotation” is the length of rotation practice reported in the articles for woody bioenergy crops like eucalypt, willow and poplar. “Rotation” is not necessary to equal to “Age” because some observations or harvest may be performed before or after the full rotation length.
– “Density” is the plant density expressed as number of plants per ha. When plant density is reported at both planting date and at harvest/observation date (after accounting for mortality), only the latter is recorded. Otherwise, plant density at planting date is recorded.

Attribute 6 “Yield” contains eight columns.

**Figure 2:**
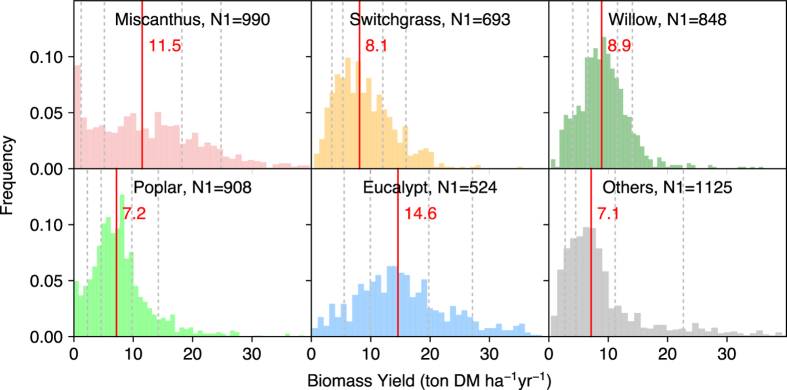
Distribution of biomass yields of different bioenergy crops in the dataset. Red vertical lines indicate the medians. The dotted lines indicate the 10, 25, 75 and 90% percentiles. N1 is the total number of entries.

**Figure 3:**
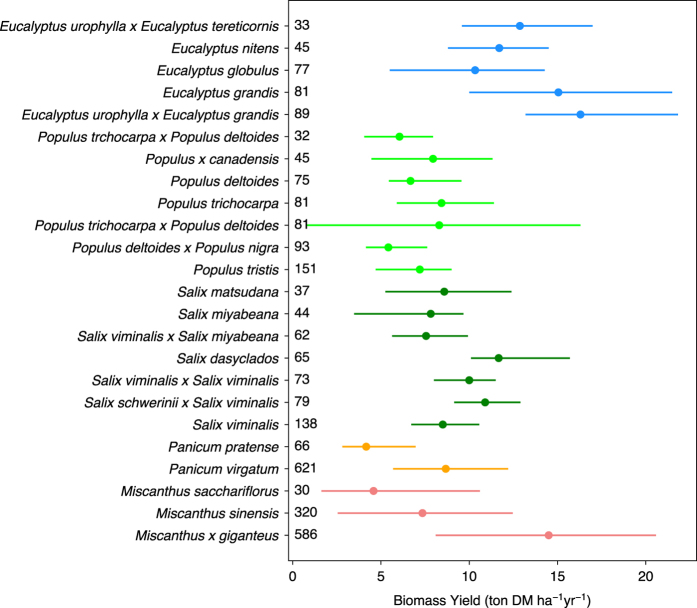
Biomass yields of main species of the five bioenergy crops in the dataset. Dots and error bars indicate the median and interquartile range. Different colors represent different bioenergy crop types. The numbers are number of entries (N1) for each species. Note that only species with N1≥30 are shown.

**Figure 4:**
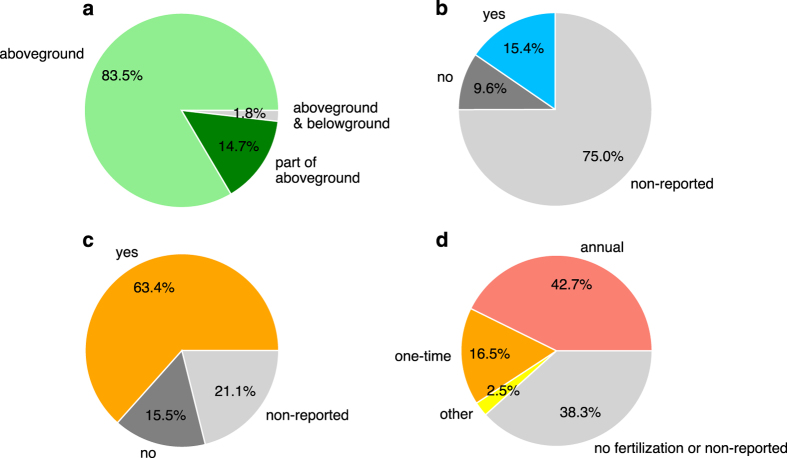
Percentages of yield data with information on biomass type and management. (**a**–**d**) are specific biomass pools constituting the yield, irrigation, fertilization, and fertilizing frequency, respectively. See “Yield_type_Index”, “Irrigation_flag”, “Fertilization_flag”, and “Fertilizing_frequency” (annual, one-time or total amount during the whole observation period) in Table 2 for details.

– Bioenergy crop yield (column “Yield”) is the main data record in this dataset. “Yield” corresponds to the mean annual harvestable biomass production. For example, if the original literature reported the total harvested biomass of poplar at a certain age, the total biomass amount is divided by age to get the mean annual biomass yield. If the original literature reported the annual harvested biomass of *Miscanthus* for several years, each annual yield is taken as one observation. The biomass yields of different bioenergy crop species show contrasted distribution patterns (Fig. 2). Compared to the other crops, biomass yields of *Miscanthus* seem to distribute more evenly. The medians of the five main bioenergy crops are ordered as follows (Fig. 2): eucalypt > *Miscanthus* > willow > switchgrass > poplar. The median value of other bioenergy crops (category “Others” in Fig. 2) is similar to that of poplar. Biomass yields of main species of the five bioenergy crops types are also in Fig. 3. Different species from the same crop type also show different yields.
– The units of yields have all been converted into ton DM ha^-1^ yr^-1^ (“Unit”).
– Some studies also reported the uncertainties of their observations (e.g. standard errors), which are recorded in the columns “Error” and “Error_type”.
– “Yield_type” is the corresponding biomass part being harvested (e.g. aboveground, stem or stem plus branches) to the “Yield”. It is detailed as that reported in the original articles.
– We classified “Yield_type” into three categories (“Yield_type_Index”): aboveground biomass, most of aboveground biomass but not all (e.g. only stem or stem plus branches), and the total of aboveground and belowground biomass. Biomass yields are reported as aboveground biomass in most studies (Fig. 4a), and the percentages of yield data in each category are 83.5, 14.7 and 1.8%, respectively.
– “Yield_estimation” is the method to measure / estimate the biomass yields used in the original study. For example, biomass yield can be weighted after harvest and drying or estimated using allometric equations (especially for some woody crops).
– “Yield_origin” documents the conversion of original reported values to the yield in the dataset. Information like “directly reported”, “averaged by Age”, “multiplied by Density” or “unit conversion” is recorded in this column.

Attribute 7 “Management” contains twelve columns documenting the management practices, mainly irrigation and fertilization.

**Figure 5:**
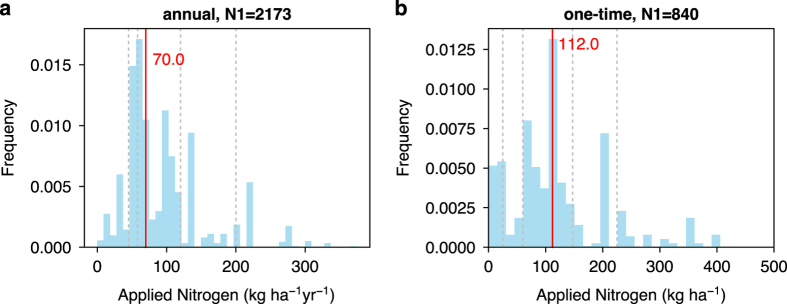
Distribution of nitrogen fertilizer applied in the dataset. (**a**) shows the distribution of the amount of nitrogen applied annually (“Fertilizing_frequency” = ”annual”) and (**b**) is for nitrogen applied only one-time (“Fertilizing_frequency” = ”one-time”). N1 is the total number of entries. Red vertical lines indicate the medians. The dotted lines indicate the 10, 25, 75 and 90% percentiles.

– “Management” is the descriptive notes of managements reported in the original articles. It mainly includes the managements that are difficult to quantify like application of manure, pesticides, and herbicide, or some applied fertilizer or irrigation for which precise applied amount is unknown.
– “Irrigation” documents the irrigated amount in mm yr^-1^ or a descriptive note when it is difficult to be converted into mm yr^-1^ (e.g. “2-6 cm/week during growing season”).
– A flag (“Irrigation_flag”) was added to indicate each entry irrigated or not. Note that some watering is sometimes only applied at plantation or during the first year of establishment to help the plant survive. In this case, if no irrigation is further applied, the “Irrigation_flag” is set to be “no”. Overall, 15.4% of the entries are labeled as “yes” (irrigation) in “Irrigation_flag” (Fig. 4b).
– Applied fertilizers are detailed in the amount (kg ha^-1^) in “Nitrogen”, “Phosphorus”, “Potassium”, “Calcium”, “Magnesium” and “Boron”. If there are other fertilizers reported, it is recorded in “Other_fertilization” in terms of fertilizer name and amount.
– “Fertilization_flag” was added to filter entries with/without fertilization. Contrary to irrigation, fertilization applied at plantation or during the first year of growth is flagged as “yes” (fertilized). Based on “Fertilization_flag”, 63.4% of entries are fertilized to some extent (Fig. 4c).
– “Fertilizing_frequency” records the frequency of fertilizer applied (mainly for nitrogen application). Fertilizer is applied annually in 42.7% of the total entries (Fig. 4d), and in this case applied fertilizer amount refers to annual rate. 16.5% of the total entries have one-time fertilizer application during the observation period (Fig. 4d), and in this case applied fertilizer amount refers to the total amount. There are also 2.5% of entries that have multiple/irregular fertilizer applications (Fig. 4d), and the total amount during a certain period is recorded.
– The distributions of applied nitrogen fertilizer are shown in Fig. 5. The median values of nitrogen applied annually (Fig. 5a) and one-time over the observation period (Fig. 5b) are 70 kg ha^-1^ yr^-1^, and 112 kg ha^-1^, respectively.

In some columns, there are cells without values, which mean that the information was not available or not reported in the original articles.

## Technical Validation

Each original article was carefully read at least twice, and special attentions were paid to the values of biomass yields and fertilization information. While compiling the dataset, detailed notes were added in the file to document all data that were adjusted/extrapolated from the original articles. For example, we added “Google map” and “center of reported area” in the column “Coordinate_origin” to document how the latitude and longitude were derived. We also added “Yield_origin” column to record the conversion of the original values to the yield values in the dataset, like unit conversion, averaged by age or multiplied by plantation density. This helps to track and check how the data were compiled. After the data extraction, the data records were checked throughout against their corresponding original articles. We used “Python Data Analysis Library — pandas” (version 0.20.0, http://pandas.pydata.org/) to perform a systematical examination and provided an evaluation report (Supplementary File 1) with basic information (e.g. unique values, value counts) and statistics (e.g. mean, interquartile values). The formats of each column (numerical or string) were checked to correct the mistyping in the numerical columns like “Yield” and “Rotation”. We also made visualization of data distribution for the columns that contain numeric values (e.g. spatial maps for “Latitude” and “Longitude”, frequency distributions for “Rotation”, “MAT” and “MAP”) and manually checked the outliers by validating them in the original articles. Especially for the latitude and longitude of each site, we carefully checked the consistency between the location and country names and manually validated the inconsistent ones (see the evaluation report, Supplementary File 1). Some sites fall in the sea based on the reported coordinates probably because of the precision of values since these sites are in the coastal area. For these sites, we corrected slightly the longitude and latitude to the near land (see the evaluation report, Supplementary File 1). For “Yield” and other types of quantitative data, we also plotted the frequency distribution for each of the considered bioenergy crop species (Fig. 2, Fig. 3 and Fig. 5) and returned to the original articles for checking extreme values.

## Usage Notes

This global dataset includes a great number of yield data for major lignocellulosic bioenergy crop species. It could be potentially used in future meta-analyses to identify the most productive species in contrasted environments, and to study the effect of various factors impacting bioenergy crop biomass production like genetic types, plant density, irrigation and fertilization, or the interactions between different factors. Such meta-analyses have already been conducted but with a much smaller number of data^10^. In addition, by coupling our dataset with weather databases, it will be possible to establish relationships between biomass yield and climatic conditions using statistical models. Such relationships could then be used to predict the global biomass production based on high-resolution climate observation data (e.g. Climate Research Unit dataset^21^, CRU).

The dataset is also valuable for the evaluation of bioenergy crop yields used in IAMs or simulated by global vegetation models. In IAMs, bioenergy deployment is analyzed through a cost-benefit framework based on biomass yields simulated by global vegetation models^18–20^. One output of IAMs is future land use maps that describe the fraction of each cultivated vegetation type, including bioenergy crops. In periodical climate change assessments conducted by e.g. the Intergovernmental Panel on Climate Change (IPCC), those land use maps are used as inputs to spatially explicit grid-based vegetation models run stand-alone or part of coupled earth system models (ESMs) to simulate the details of the terrestrial carbon and nutrients cycle and its evolution in response to increasing CO_2_ and variable climate. Thus, the performance of bioenergy crop modelling in global vegetation models is critical to determine the interactions between ecosystem and climate. As coordinates are available for all yield entries, it is possible to compare the outputs of global vegetation models to the recorded yield data. But this kind of model assessment should be done with caution: (i) Harvested biomass vs. crop residuals left in fields need to be carefully distinguished when using yield data to evaluate model outputs. For example, models simulating aboveground biomass should be evaluated with yield data corresponding to “aboveground” or “part of aboveground” in “Yield_type_Index” (Table 2). (ii) Management techniques considered in vegetation models should be consistent with those of yield data. For example, if a model aims at simulating biomass production without fertilizer and irrigation, only yield data without irrigation and fertilization should be considered for model assessment. Similarly, if a model simulates biomass production at different fertilizer application levels, these levels should be consistent with yield data used for model assessment. (iii) There are some data gaps in several regions (e.g. Africa and Russia, Fig. 1 and Table 1), and model assessment could not be conducted in these regions. However, as new experiments are conducted every year, we may expect that our dataset could be updated in the future for the same species or even for new species.

## Additional information

**How to cite this article**: Li, W. *et al*. A global yield dataset for major lignocellulosic bioenergy crops based on field measurements. *Sci. Data* 5:180169 doi: 10.1038/sdata.2018.169 (2018).

**Publisher’s note**: Springer Nature remains neutral with regard to jurisdictional claims in published maps and institutional affiliations.

## Supplementary Material



Supplementary Information

## Figures and Tables

**Table 1:** Numbers of yield data (N1) and of sites (N2) for different bioenergy crop types in different regions.

	**All**		***Miscanthus***		**Switchgrass**		**Willow**		**Poplar**		**Eucalypt**		**Others**	
**Continent**	**N1**	**N2**	**N1**	**N2**	**N1**	**N2**	**N1**	**N2**	**N1**	**N2**	**N1**	**N2**	**N1**	**N2**
North America	99	99	14	14	39	39	15	15	29	29	6	6	826	25
South America	114	23	0	0	0	0	0	0	0	0	105	23	9	2
Europe	1949	124	805	60	76	4	494	28	378	45	8	1	188	11
Africa	8	3	0	0	0	0	0	0	0	0	8	3	0	0
Asia	509	75	9	3	59	8	34	9	158	27	176	29	73	8
Oceania	218	31	0	0	0	0	1	1	1	1	187	30	29	5
Globe	5088	355	990	77	693	51	848	53	908	102	524	92	1125	51
Note that N2 for “All” is not equal to the sum of N2 over all crops because several measurements are sometimes reported for different crop types in a given site.														

**Table 2:** Description of attributes and columns in the dataset.

**Attribute**	**Column #**	**Column Name**	**Unit**	**Note**
1. Reference	1	Reference	-	Author names and publication year (plus journal initials for a distinction when needed).
2. Site Location	2	Country	-	Country where the field site locates.
	3	Site	-	Name of site (e.g. state, city, county…).
	4	Coordinate_origin	-	Origin of coordinate (reported site, Google map or center of reported area).
	5	Latitude	Degree North/South	Latitude of field site.
	6	Longitude	Degree East/West	Longitude of field site.
	7	Elevation	m a.s.l.	Elevation of field site.
3. Climate	8	Temperature	˚C	Mean annual temperature (MAT) at field site.
	9	Rainfall	mm yr^-1^	Mean annual precipitation (MAP) at field site.
4. Soil	10	Clay	%	Clay fraction in the soil at field site.
5. Plantation	11	Field_type	-	Fied type of measurements (experimental trial, farmer's field or natural field).
	12	Field_size	-	Size of the whole field or sampling plot.
	13	Crop_type	-	Bioenergy crop types: “Eucalyptus”, “Poplar”, “Willow”, “Miscanthus”, “Switchgrass” and others (in common names).
	14	Species	-	Species names of bioenergy crops.
	15	Detailed_species_information	-	Species names with other detailed information (e.g. genotypes) of bioenergy crops.
	16	Planting_date	-	Bioenergy crop planting date.
	17	Harvest_year	-	Harvest year corresponding to the reported yields.
	18	Harvest_date	-	Time (ususally month) of harvest.
	19	Age	yr	Age of bioenergy crops at harvest or observation.
	20	Rotation	yr	Length of rotation.
	21	Density	plants ha^-1^	Planting density.
6. Yield	22	Yield	see "Unit"	Yield or biomass increment per year.
	23	Unit	-	Unit of “Yield” and “Error”, all converted into “ton DM ha^-1^ yr^-1^”
	24	Error	see "Unit"	Reported measurement errors.
	25	Error_type	-	Type of “Error” (e.g. standard deviation or standard error).
	26	Yield_type	-	Biomass part (e.g. aboveground, stem only or stem plus branches) corresponding to “Yield”.
	27	Yield_type_Index	-	Groups of Yield_type, including aboveground, part of aboveground (e.g. stem only), and total of aboveground and belowground.
	28	Yield_estimation	-	Methods of yield estimation in the original article (e.g. dried, weighted, allometric equation).
	29	Yield_origin	-	Conversion of original values to yield in the dataset (e.g. unit conversion, averaged by Age).
7. Management	30	Management	-	Description of managements.
	31	Irrigation	- or mm yr^-1^	Irrigated or not; irrigation amount is reported in case of irrigation.
	32	Irrigation_flag	-	A flag to indicate irrigation (yes, no or NaN).
	33	Fertilization_flag	-	A flag to indicate fertilization (yes, no or NaN).
	34	Fertilizing_frequency	-	A flag to indicate fertilizing frequency (mainly for nitrogen application): applied annual, thus annual amount; applied only once during the period, thus the total amount; total application amount during the period; NaN, no fertilization or non-reported.
	35	Nitrogen	kg ha^-1^	Applied fertilizer amount.
	36	Phosphorus	kg ha^-1^	Applied fertilizer amount.
	37	Potassium	kg ha^-1^	Applied fertilizer amount.
	38	Calcium	kg ha^-1^	Applied fertilizer amount.
	39	Magnesium	kg ha^-1^	Applied fertilizer amount.
	40	Boron	kg ha^-1^	Applied fertilizer amount.
	41	Other_fertilization	-	Other types of fertilizer applied.
