# Supplementary material for: A global yield dataset for major lignocellulosic bioenergy crops based on field measurements
Source: Sci Data. 2018 Aug 21;5:180169. doi: 10.1038/sdata.2018.169 (PMC6107052; doi:10.1038/sdata.2018.169)
Supplement: Supplementary Information [file sdata2018169-s2.pdf]

# Evaluation Report

## Table of Contents


**1 Load python package and dataset** **1**

**2 Basic information of dataset** **1**

**3 Detailed information check** **3**

**3.1 Locations** **3**

3.1.1 Country, Site, Coordinate_origin, Lat, Lon, Elevation 3

3.1.2 Check Lat, Lon in the correct country and on the land 8

**3.2 Climate and Soil** **12**

**3.3 Field information** **13**

3.3.1 Field_type, Field_size, Crop_type 13

3.3.2 Crop_type, Species, Detailed_species_information 15

3.3.3 Planting_date, Harvest_year, Harvest_date, Age, Rotation, Density 30

**3.4 Yield** **31**

3.4.1 Yield, Unit, Error, Error_type 32

3.4.2 Yield_type, Yield_type_Index 32

3.4.3 Yield_estimation, Yield_origin 34

**3.5 Management** **34**

3.5.1 Management 35

3.5.2 Irrigation, Irrigation_flag 36

3.5.3 Fertilization_flag, Fertilizing_frequency, Nitrogen, Phosphorus, Potassium, Magnesium, Boron, Calcium, Other_fertilization 37

# py03a_data_evaluation

June 21, 2018

## 1 Load python package and dataset

```
In [1]: # load data
        %matplotlib inline
        import pandas as pd
        import numpy as np
        import warnings; warnings.simplefilter('ignore')

        #===========================================================================
        # This script documents the basic information and statistics of
        # the bioenergy crop yield dataset.
        # It can be run using Python with packages of "pandas", "numpy" and "osgeo".
        # For the checking of consistentcy between coordinates and country names
        # (Section 3.1.2),
        # we also used a country border map from World Borders Dataset, which can
        # be downloaded from
        # http://thematicmapping.org/downloads/world_borders.php
        #
        # Contact: wei.li@lsce.ipsl.fr
        # 20 Jun 2018
        # ==========================================================================

        # load bioenergy yield dataset
        #*********Change to the file name of the dataset************
        df1 = pd.read_excel('Field_sites_180329_sub.xlsx', 'Data',
                            index_col=None, na_values=['NA'])
```

## 2 Basic information of dataset

```
In [2]: # Basic information
        ndf1 = len(df1)
        print 'Number of entries:', len(df1)
        print 'Number of columns:', len(df1.columns)
        print 'Column names:'
        for i in range(len(df1.columns)):
          print 'Column #%d'%(i+1), df1.columns[i]
```

```
Number of entries: 5088
Number of columns: 41
Column names:
Column #1 Reference
Column #2 Country
Column #3 Site
Column #4 Coordinate_origin
Column #5 Latitude
Column #6 Longitude
Column #7 Elevation
Column #8 Temperature
Column #9 Rainfall
Column #10 Clay
Column #11 Field_type
Column #12 Field_size
Column #13 Crop_type
Column #14 Species
Column #15 Detailed_species_information
Column #16 Planting_date
Column #17 Harvest_year
Column #18 Harvest_date
Column #19 Age
Column #20 Rotation
Column #21 Density
Column #22 Yield
Column #23 Unit
Column #24 Error
Column #25 Error_type
Column #26 Yield_type
Column #27 Yield_type_Index
Column #28 Yield_estimation
Column #29 Yield_origin
Column #30 Management
Column #31 Irrigation
Column #32 Irrigation_flag
Column #33 Fertilization_flag
Column #34 Fertilizing_frequency
Column #35 Nitrogen
Column #36 Phosphorus
Column #37 Potassium
Column #38 Calcium
Column #39 Magnesium
Column #40 Boron
Column #41 Other_fertilization
```

# 3 Detailed information check

## 3.1 Locations

**Country, Site, Coordinate_origin, Latitude, Longitude, Elevation**

### 3.1.1 Country names, number of site, Coordinate_origin, Elevation, Site names

```
In [46]: # Locations
         print 'Number of countries:', df1.Country.nunique()
         country = np.sort(df1.Country.unique())
         print 'Country names, number of entries:'
         for tmp in country:
           print '  ', tmp, np.sum(df1.Country==tmp)

         df1['LatLon'] = pd.Series(np.zeros(ndf1), index=df1.index)
         for i in np.arange(ndf1):
             df1.loc[i, 'LatLon'] = '%.2f %.2f'%(df1.loc[i, 'Latitude'], df1.loc[i, 'Longitude
         print '-'*80
         print 'Number of sites at 0.01 deg precision:', df1.LatLon.nunique()
         print '-'*80
         print 'Coordinate_origin, number of sites:\n', df1.Coordinate_origin.value_counts()
         print '-'*80
         print 'Elevation, statistical description:\n', df1.Elevation.describe()
```

```
Number of countries: 31
Country names, number of entries:
   Australia 167
   Austria 40
   Belgium 61
   Brazil 114
   Canada 176
   China 436
   Croatia 7
   Czech Republic 22
   Denmark 85
   Estonia 6
   Finland 4
   France 189
   Germany 227
   Greece 10
   India 44
   Iran 15
   Ireland 21
   Italy 291
   Japan 14
   Latvia 6
   Netherlands 2
   New Zealand 51
```

```
   Poland 80
   Portugal 54
   South Africa 7
   Sweden 136
   Switzerland 2
   Turkey 15
   Uganda 1
   United Kingdom 691
   United States 2114
--------------------------------------------------------------------------------
Number of sites at 0.01 deg precision: 384
--------------------------------------------------------------------------------
Coordinate_origin, number of sites:
reported site             4167
Google map                 849
center of reported area     72
Name: Coordinate_origin, dtype: int64
--------------------------------------------------------------------------------
Elevation, statistical description:
count    2187.000000
mean      225.584324
std       280.953534
min        -3.000000
25%        35.700000
50%       110.000000
75%       300.000000
max      1750.000000
Name: Elevation, dtype: float64
```

```
In [47]: # check Column 'Site'
         print np.sort(df1.Site.unique())
```

```
[nan u'10 km southeast of Falkenberg'
 u'ADAS Arthur Rickwood, Cambridgeshire'
 u'ADAS Arthur Rickwood, Cambridgeshire; ADAS Drayton, Warwickshire'
 u'ADAS Boxworth, Cambridgeshire' u'ADAS Bridgets, Hampshire'
 u'ADAS Gleadthorpe, Nottinghamshire' u'ADAS High Mowthorpe'
 u'ADAS Rosemaund, Herefordshire' u'Abbachhof' u'Aberystwyth, Ceredigion'
 u'Aberystwyth, Wales' u'Alice Holt, Hampshire' u'Allensbush' u'Ampermoos'
 u'Ansai Research Station, Chinese Academy of Science, Shaanxi'
 u'Ansai, Yanan, Shaanxi' u'Aokautere, Palmerston North'
 u'Appling soil, Lunenberg County, VA' u'Aracruz, ES'
 u'Aracruz, Espirito Santo' u'Ardon' u'Argyll, Scotland' u'Arlington, WA'
 u'Ascheberg' u'Ashland WI' u'Atzembrugg' u'Atzenbrugg' u'Baoying, Jiangsu'
 u'Bedford County, PA' u'Beeville, Texas'
 u'Beipo Linchang, Zhanjiang, Guangdong' u'Bigarello' u'Bocaiuva, MG'
 u'Bodarna' u'Boisbriand, QC' u'Boitzenhagen' u'Boola, Gippsland, Victoria'
```

u'Boom, Antwerpen' u'Braintree, Essex' u'Bridport, Tasmania'
u'Brimley, MI' u'Brookings, SD' u'Brotas, Sao Paulo'
u'Brownstown, Brownstown Research and Education Center, Brownstown, IL'
u'Bruner Fram of the Agronomy and Agricultural Engineering Research center, Ames, Iowa'
u'Buckfast Abbey, Devon' u'Buckland, Tasmania' u'Bullstofta'
u'Bush, Midlothian' u'Bystrice' u'Cambridgeshire'
u'Cann River, East Gippsland, Victoria' u'Canstein' u'Caramagna Piemonte'
u'Carrajung, Victoria'
u'Carrington irrigated, Carriton Research and Extension Center, Carrington'
u'Carteret, North Carolina' u'Casale Monferrato' u'Catania, Sicily'
u'Cavallermaggiore' u'Chilliwack, BC' u'Cloquet, MN' u'Colchester, Essex'
u'Collamer soil, Ithaca, NY' u'Collamer-e soil, Ithaca, NY'
u'Companhia Vale do Rio Doce (CVRD) forest reserve' u'Constableville, NY'
u'Danish Institute of Agricultural Sciences, Research Center Foulum, Jutland'
u'Danzhou, Hainan' u'Darda Forest Office' u'Darkan, Western Australia'
u'Davidson soil, Orange County, VA'
u'Dekalb, Northern Illinois Agronomy Research Center, Shabbona, IL'
u'Delfzijl'
u'Dixon Springs, Dixon Springs Agricultural Research Center, Simpson, IL'
u'Domaninek' u'Donauried' u'Dongmen Forestry, Guangxi'
u'Dongmen Linchang, Guxangxi' u'Dongshanqiao, Nanjing'
u'Dongying, Shandong' u'Dumersheim, Upper Rhine Valley'
u'Durmersheim, Upper Rhine Valley'
u'E.V. Smith Research Center, Shorter, AL'
u'E.v. Smith Research Center, Plant Breeding Unit, Shorter, Alabama'
u'Eazma Forest Office' u'Ehrendorf' u'Elblag'
u'Emile A. Lods Research Centre, McGill University, Montreal' u'Enkoping'
u'Enna'
u'Enrico Avanzi Interdepartmental Centre for Agro-Environmental Research (CIRAA) of the Univer
u'Entr-Rios' u'Erie Co., Geophyta, Ohio'
u"Erie soil, Hector Nat'l. Forest, NY" u'Erie soil, Ithaca, NY'
u'Escanaba, MI' u'Eunapolis, BA'
u'Experimental Farm of the University of Thessaly, Velestino, Volos'
u'FAL Braunschweig, Boitzenhagen, Niedersachsen'
u'Fairfield, Wayne County Extension Office, Fairfield, IL' u'Fairmont, MN'
u'Fargo, ND'
u'Felin Experimental Fram, University of Life Science, Lublin'
u'Fengshan, Xiaodian, Suqian' u'Fichtel mountain'
u'Forest Hills, Wagga Wagga, NSW'
u'Forest Research Station, Madahally, Karnataka'
u'Foulumgard, Aarhus University, Foulum' u'Fredonia, NY' u'Friemar'
u'Fusui, Guangxi' u'Ganan, Jilin' u'Gaofeng Linchang, Guangxi'
u'Gaotang, Shandong' u'Gauland' u'Gazzo Bigarello'
u'Glencoe, Gippsland, Victoria' u'Glenfield Good, Glenfield, ND'
u'Glenfield, ND' u'Gloucestershire, England'
u'Gongqing Farm Nursery, Zhenjiang, Jiangsu' u'Granite Falls, MN'
u'Grays Harbor County, Washington' u'Guan County, Shandong'
u'Guanhaes, MG' u'Gunterslebenl' u'Gutenzel, Upper Swabia'

u'Gutenzell, Upper Swabia' u'Hallertau' u'Hamakua coast, Hawaii'
u'Hamakuapoko Experimental Site, Maui, Hawaii'
u'Hangyun Linchang, Baoying, Jiangsu'
u'Hanyuan Forestry Farm, Baoying, Jiangsu'
u'Harshaw Forestry Research Farm, Rhinelander'
u'Havana, Central Illinois Irrigated Growers Association, Havana, IL'
u'Headley' u'Hechuan, Guyuan, Ningxia' u'Heilongjiang'
u'Heishui Linchang, Jianping, Liaoning' u'Hepu, Guangxi'
u'Hetou Linchang, Leizhou, Haihan'
u'Hettinger Research and Extension Center at Hettinger, ND'
u'Hilo, Hawaii' u'Hilton, Bridgetown, Western Australia'
u'Honeoye-e soil, Geneva, NY' u'Hornum Research Station, Jutland'
u'Horqin, Inner Mongolia'
u'Horticultural Research Station, Aokautere, Palmerston North'
u'Hosakote Forest Station, Karnataka' u'Huaibei, Anhui' u'Huaining, Anhui'
u'Huaisuo, Dahe, Sandushuizu county, Guizhou'
u'Hugo Sauer Nursery , Forestry Science Laboratory in Rhinelander'
u'Hugo Sauer Nursery, Rhinelander, Wisconsin'
u'INRA experimental station, Estrees-Mons' u'INRA experimental unit, Mons'
u'INRA, Orleans' u'IPF PAS, Poznan'
u'ISU McNay Research Farm, Chariton, Iowa' u'Ihinger Hof'
u'Ihinger Hof of the University of Hohenheim'
u'Ihinger Hof, near stuttgart' u'Ilz' u'Index, WA' u'Innertavle'
u'Institut fur Agrartechnik Bornim, Potsdam'
u'Institute for Agricultural and Fisheries Research (ILVO), Melle'
u'Institute for Sustainable Agro-ecosystem Services, Graduate School of Agricultural and Life
u'Instructional Farm, ASPEE College of Horticulture and Forestry, Navsari Agricultural Univers
u'International Paper site, Mogi Guacu, Sao Paulo' u'Invergowrie'
u'Itatinga experimental station' u'Jiangxia, Hubei' u'Johannesholm'
u'Jyndevad' u'Jyndevad Experimental Station, Southern Jutland' u'Kadesjo'
u'Karamay, Xinjiang' u'Karinslund' u'Karinslund, Skane County' u'Karkloof'
u'Kendaia soil, Aurora, NY' u'Kilkenny' u'Kirchberg' u'Klein Markow'
u'Knoxville, TN' u'Koping' u'Kwazulu-Natal Midlands'
u'LFA Gulzow, Klein Markow, Mecklenburg-Vorpommern'
u'LWG Veitshochheim, Guntersleben-Klaranlage, Bayern'
u'LWG Veitshochheim, Guntersleben-Volkenschlag, Bayern'
u'Langaveka experimental field station'
u'Leika, Dongmen Linchang, Guangxi' u'Leizhou, Guangdong'
u'Lenoir, North Carolina' u'Leonard, ND' u'Lilla Fagerhult'
u'Linan, Zhejiang' u'Lingao, Hainan' u'Lombriasco' u'Long Ashton, Avon'
u'Long Ashton, Somerset' u'Longview, OR' u'Loughgall, Northern Ireland'
u'Lower Coastal Plains Substation, Camden, AL' u'Luis Antonio, SP'
u'Madalin soil, Willsboro, NY' u'Malins Hed' u'Manawatu River, Dannevirke'
u'Mangakino, central North Island' u'Manjimup, Western Australia'
u'Mardin soil, Freeville, NY' u'Markgrafneusiedl'
u'Markington, North Yorkshire' u'Maryvale, Gippsland, Victoria'
u'Mepal, Norfolk' u'Meridian Seed Orchard, Olympia, Washington'
u'Miaofengshan, Beijing' u'Middlebury, VT' u'Midlothian, Scotland'

u'Milaca, MN' u'Mira'
u"Missouri river floodplain at the University of Missouri's Horticulture and Agroforestry Rese
u'Mogi Guacu, SP' u'Mogi Guacu, Sao Paulo' u'Mondovi, WI' u'Monroe, WA'
u'Montreal Botanical Garden, Terrebonne' u'Morris, MN'
u'Mount Vernon, Washington'
u'Mountain Horticultural Crops Research Station, Mills River, North Carolina'
u'Mummbalup, Western Australia' u'Muzizi Tea Estate, Amuru'
u'NE-facing Cecil soil, Amelia County, VA'
u'Nangeng, Haikou, Anqing, Anhui' u'Nanjing, Fujian'
u'Napoleons, Ballarat, Victoria' u'Nasbyholm' u'Niedere Geest'
u'Nisqually, WA'
u'North Central Florida, U. Florida Plant Science Research and Education Unit'
u'Northcliffe,  Western Australia' u'Nunamara'
u'Orr, Orr Research and Education Center, Perry, IL' u'Orsay'
u'Orting, WA' u'Ottawa Co., Geophyta, Ohio' u'Paimio'
u'Paiyangshan Linchang, Ningming, Guangxi' u'Pemberton' u'Picardie'
u'Piedmont Substation' u'Piedmont Substation, Camp Hill, AL' u'Pierre, SD'
u'Pingguo, Guangxi' u'Pingsha, Jinwan, Zhuhai' u'Pisa'
u'Pitteater Research Station, Hobart'
u'Plant Science Research and Education Unit, Citra, FL'
u'Pointe-aux-Prairies Park, Montreal' u'Pubei, Guangxi'
u'Pujiangzhen, Minhang, Shanghai' u'Pustnas' u'Puyallup, Washington'
u'Qingyang, Gansu' u'Qinzhou' u'Qiongzhong, Hainan'
u'Qipo Linchang, Nanning, Guangxi' u'Quinta do Furadouro, Obidos'
u'Red River Valley of the North at Prosper, ND'
u'Research Unit for Cropping Systems in Dry Environments, Rutigliano'
u'Research farm of College of Agriculture, University of Tehran, Karaj'
u'Retford, Nottinghamshire' u'Rhine valley'
u'Rhinebeck soil, Willsboro, NY' u'Rockport, WA' u'Roslin, Bush Estate'
u'Rothamsted' u'Rothamsted Experimental Station Farm' u'Rothamsted Farm'
u'Rottaia Field Experimental Station, Pisa' u'Rydsgard'
u'SW-facing Cecil soil, Amelia County, VA' u'Saare' u'San Paulo'
u'Sand Mountain substation, Crossville, AL'
u'Sandusky Co., Geophyta, Ohio' u'Sangletorp' u'Santiam, OR'
u'Saskatoon, SK' u'Savoy, IL' u'Sayward and Campbell River, BC' u'Scania'
u'Shandong' u'Shiling Linchang, Lianjiang, Guangdong' u'Sichuan'
u'Silver Creek, Morwell, Victoria' u'Sioux Falls, SD' u'Skandia, MI'
u'Smilkov' u'Snake Valley, Ballarat, Victoria' u'Snoqualmie, WA'
u'South Farms, University of Illinois at Urbana-Champaign'
u'South Molton, Devon' u'St. Florian'
u'State University of New York, College of Environmental Science and Forestry, genetics field
u'Steinbrunn' u'Stephenville, Texas' u'Stillwater, Oklahoma' u'Storrs, CT'
u'Sturup' u'Suffolk, England' u'Sulingen' u'Sumner, Washington'
u'Suzano, Teixeiras de Freitas, Bahia' u'Tanikon' u'Tarai belt, Himalayan'
u'Tasmania' u'Teixeira de. Freitas, BA'
u'Terrestrial Ecology, Bush Estate, Midlothian '
u'Texas A&M University Agricultural Research and Extension Centers, Dallas, TX'
u'Texas A&M University Agricultural Research and Extension Centers, Stephenville, TX'

```
u'Tianjin' u'Tibet' u'Tim Shea' u'Tipperary'
u'Toa Baja experiment station, University of Puerto Rico'
u'Tobacco Research Station, Oxford, North Carolina' u'Toolangi, Victoria'
u'Toolara Forest Reserve, Gympie, Queensland' u'Torup'
u'Tree Improvement Center, Olympia, Washington'
u'Tres Marias, Minas Gerais' u'Trier' u'Tully, NY' u'Tully, New York'
u'Tully, New York, experiment #3' u'Tulou, Machikou, Changping, Beijing'
u'UF Everglades Research and Education Center, Belle Glade, FL'
u'UF Plant Science Research and Education Unit, Citra, FL'
u'UF Range Cattle Research and Education Center, Ona, FL'
u'Ultuna, Uppsala'
u'University Farm, Haryana Agricultural University, Hisar'
u'Upper Coastal Plains Substation, Winfield, AL'
u'Upper St. Lawrence region, Montreal'
u'Urbana, Crop Science Research and Education Center, Urbana, IL'
u'Valpovo Forest office' u'Veracel, Bahia' u'Veracel, Eunapolis, Bahia'
u'Vinovo'
u'Waimanalo Research Station of the University of Hawaii, Hawaii'
u'Waimanalo, Oahu, Hawaii' u'Wanqiong, Hainan' u'Wantaixing, Eerduosi'
u'Waseca, MN' u'Weidu Linchang, Guangxi' u'Weinfelden' u'Westfield'
u'Willington, Bedfordshire' u'Woburn' u'Writtle College farm, Essex'
u'Wuming, Guangxi' u'Xiaotangshan, Changping, Beijing'
u'Ximagezhuang, Daxing, Beijing' u'Xinjiang' u'Xuejiazhuang, Shanxi'
u'Yanchi, Ningxia' u'Yangling, Shaanxi' u'Yendon, Ballarat, Victoria'
u'Yinan County, Shandong' u'Yingxian, Shanxi'
u'Yuanzhongchang, Jinzhou, Liaoning' u'Zixing, Hunan'
u'Zongyang Dyke, Anhui' u'campus of Selcuk University in Konya'
u'central Scotland' u'central and western part of Latvia'
u'experimental center of Rottaia Pisa' u'ter Apel'
u'the University of Illinois Energy Farm, Urbana, Illinois']
```

### 3.1.2 Check Latitude, Longitude in the correct country and on the land

In [48]:
```python
#---define a function to convert coordinate to country name
from osgeo import ogr
# country borders are downloaded from World Borders Dataset,
# http://thematicmapping.org/downloads/world_borders.php
driver = ogr.GetDriverByName('ESRI Shapefile')
country_file = driver.Open('TM_WORLD_BORDERS_SIMPL-0.3.shp')
def Coor_Country(lat, lon):
  point = ogr.Geometry(ogr.wkbPoint)
  point.AddPoint(lon, lat)
  layer = country_file.GetLayer()
  country_name = 'None'
  for i in np.arange(layer.GetFeatureCount()):
    country_shp = layer.GetFeature(i)
    if country_shp.geometry().Contains(point):
```

```
            country_name = country_shp.GetField('NAME')
        return country_name
```

In [49]:
```
#----get the country name from lat and lon of each entry
Country_check = np.array([])
for i in np.arange(ndf1):
  lat, lon = df1.Latitude[i], df1.Longitude[i]
  Country_check = np.append(Country_check, Coor_Country(lat, lon))
np.save('smy03a_Country_check.npy', Country_check)

# Country_check = np.load('smy03a_Country_check.npy')
# replace 'Iran (Islamic Republic of)' with 'Iran'
Country_check[Country_check=='Iran (Islamic Republic of)'] = 'Iran'
Country_check_uni = np.unique(Country_check)
print Country_check_uni
print country
print len(Country_check_uni), len(country)
# Portugal
```

```
['Australia' 'Austria' 'Belgium' 'Brazil' 'Canada' 'China' 'Croatia'
 'Czech Republic' 'Denmark' 'Estonia' 'Finland' 'France' 'Germany' 'Greece'
 'India' 'Iran' 'Ireland' 'Italy' 'Japan' 'Latvia' 'Netherlands'
 'New Zealand' 'None' 'Poland' 'Portugal' 'South Africa' 'Sweden'
 'Switzerland' 'Turkey' 'Uganda' 'United Kingdom' 'United States']
[u'Australia' u'Austria' u'Belgium' u'Brazil' u'Canada' u'China' u'Croatia'
 u'Czech Republic' u'Denmark' u'Estonia' u'Finland' u'France' u'Germany'
 u'Greece' u'India' u'Iran' u'Ireland' u'Italy' u'Japan' u'Latvia'
 u'Netherlands' u'New Zealand' u'Poland' u'Portugal' u'South Africa'
 u'Sweden' u'Switzerland' u'Turkey' u'Uganda' u'United Kingdom'
 u'United States']
32 31
```

In [50]:
```
# to check if the countries names are consistent
for i in np.arange(len(country)):
  country_tmp = country[i]
  idxtmp1, = np.where(Country_check==country_tmp)
  idxtmp2, = np.where(df1.Country==country_tmp)
  print  country_tmp
  for j in idxtmp1:
    if j not in idxtmp2:
      print j
print '*'*100
print Country_check[range(431,439)]
print np.array(df1.Country[range(431,439)])
print '''\nIndex 431-438 already manunally checked
(close to the border between Germany and Czech Republic)!
It is correct in the dataset.'''
```

```
Australia
Austria
Belgium
Brazil
Canada
China
Croatia
Czech Republic
Denmark
Estonia
Finland
France
Germany
431
432
433
434
435
436
437
438
Greece
India
Iran
Ireland
Italy
Japan
Latvia
Netherlands
New Zealand
Poland
Portugal
South Africa
Sweden
Switzerland
Turkey
Uganda
United Kingdom
United States
**********************************************************************************************
['Germany' 'Germany' 'Germany' 'Germany' 'Germany' 'Germany' 'Germany'
 'Germany']
[u'Czech Republic' u'Czech Republic' u'Czech Republic' u'Czech Republic'
 u'Czech Republic' u'Czech Republic' u'Czech Republic' u'Czech Republic']

Index 431-438 already manunally checked
(close to the border between Germany and Czech Republic)!
It is correct in the dataset.
```

```
In [51]: #---check referecne one-by-one for the country name == 'None'
         Missed_sites = np.unique(df1.LatLon[Country_check=='None'])
         # print Missed_sites
         for i in np.arange(len(Missed_sites)):
           idx_tmp, = np.where(df1.LatLon==Missed_sites[i])
           print i+1, 'Number of entries: %d\n'%(len(idx_tmp)),
           df1.loc[[idx_tmp[0]],['Reference','LatLon']], '\n', '-'*70
         print '*'*100
         print '''All these sites are mannually checked one-by-one;
         They are all on the land and correct;
         See the detailed information below.'''
```

```
1 Number of entries: 10
2 Number of entries: 3
3 Number of entries: 6
4 Number of entries: 8
5 Number of entries: 18
6 Number of entries: 56
7 Number of entries: 11
8 Number of entries: 2
9 Number of entries: 46
10 Number of entries: 48
11 Number of entries: 8
12 Number of entries: 6
13 Number of entries: 15
14 Number of entries: 3
***********************************************************************************************
All these sites are mannually checked one-by-one;
They are all on the land and correct;
See the detailed information below.
```

```
In [52]: info_Coor_check = '''
         Possible typos of coordinates in Marsden et al., 2010;
             Corrected in the dataset.
         Reported coordinates in sea (coastal area) in Bauhus et al., 2004;
             Corrected to land.
         Reported coordinates in sea (coastal area) in Smethurst et al., 2003;
             Corrected to land.
         Correct coordinates reported in Close et al., 2004;
             Incorrect borders (coastal area) in World Borders Dataset.
         Reported coordinates in sea (coastal area) in Pinkard and Beadle, 1998;
             Corrected to land.
         Correct coordinates reported in Parrotta, 1999;
             Incorrect borders (coastal area) in World Borders Dataset.
         Correct coordinates reported in Harrington and Fownes, 1995;
```

```
    Incorrect borders (coastal area) in World Borders Dataset.
Correct coordinates reported in Xu, 2013;
    Incorrect borders (coastal area) in World Borders Dataset.
Correct coordinates reported in Sumiyoshi et al., 2017;
    Incorrect borders (coastal area) in World Borders Dataset.
Reported coordinates in sea (coastal area) in Austin et al., 1997;
    Corrected to land.
Reported coordinates in sea (coastal area) in Han et al., 2010;
    Corrected to land.
Correct coordinates reported in Tian et al., 2017;
    Incorrect borders (coastal area) in World Borders Dataset.
Reported coordinates in sea (coastal area) in Cosentino et al., 2007;
    Corrected to land.
Correct coordinates reported in Clifton-Brown et al., 2004, Lewandowski el al., 2003;
    Incorrect borders (coastal area) in World Borders Dataset.
Correct coordinates reported in Clifton-Brown et al., 2001, AJ;
    Incorrect borders (coastal area) in World Borders Dataset.
Correct coordinates reported in Angelini et al., 2005, o di Nasso et al., 2011,
    Ek&Dawson, 1976;  Incorrect borders (coastal area) in World Borders Dataset.
Read from Google map, coastal area in Murray and Harrington, 1983;
    Corrected to land.
Reported coordinates in sea (coastal area) in Lindegaard et al., 2011;
    Corrected to land.
Reported coordinates in sea (coastal area) in Christersson, 2006;
    Corrected to land.
Read from Google map, coastal area in Schwarz et al., 1994, AAB;
    Corrected to land.
Correct coordinates reported in Verwijst et al., 1996;
    Incorrect borders (coastal area) in World Borders Dataset.
Correct coordinates reported in Christersson, 1986;
    Incorrect borders (coastal area) in World Borders Dataset.
Correct coordinates reported in Grelle;
    Incorrect borders (coastal area) in World Borders Dataset.
'''
# print info_Coor_check
```

## 3.2 Climate and Soil

### Temperature, Rainfall, Clay

```
In [53]: print '-'*80
         print 'Temperature, statistical description:\n', df1.Temperature.describe()
         print '-'*80
         print 'Rainfall, statistical description:\n', df1.Rainfall.describe()
         print '-'*80
         print 'Clay, statistical description:\n', df1.Clay.describe()
```

```
--------------------------------------------------------------------------------
Temperature, statistical description:
```

```
count    1852.000000
mean       13.265572
std         5.741732
min         4.600000
25%         9.000000
50%        11.000000
75%        15.000000
max        35.600000
Name: Temperature, dtype: float64
--------------------------------------------------------------------------------
Rainfall, statistical description:
count    2579.000000
mean      972.384880
std       523.644555
min       105.300000
25%       684.000000
50%       909.000000
75%      1125.000000
max      4600.000000
Name: Rainfall, dtype: float64
--------------------------------------------------------------------------------
Clay, statistical description:
count    707.000000
mean      24.470297
std       13.506576
min        2.000000
25%       13.600000
50%       23.000000
75%       35.000000
max       65.000000
Name: Clay, dtype: float64
```

### 3.3 Field information

**Field_type, Field_size, Crop_type, Species, Detailed_species_information, Planting_date, Harvest_year, Harvest_date, Age, Rotation, Density**

#### 3.3.1 Field_type, Field_size, Crop_type

```
In [54]: print '-'*80
         print 'Field_type, value count:\n', df1.Field_type.value_counts()
         print '-'*80
         print 'Field_size, unique values:\n', df1.Field_size.unique()
```

```
--------------------------------------------------------------------------------
Field_type, value count:
experimental trial    4793
```

```
farmer's field         286
natural field            4
Name: Field_type, dtype: int64
--------------------------------------------------------------------------------
Field_size, unique values:
[u'30 m x 30 m each plot' u'7 m x 10 m each plot' nan
 u'6 m x 8 m each plot' u'45 m2 each plot' u'300 m2 each plot'
 u'87 m2 each plot' u'3 m x 4 m each plot' u'12.5 m x 70 m'
 u'7.5 m x 15 m each plot' u'9 m x 11.5 m' u'20 m x 20 m each main plot'
 u'10 m x 10 m each plot' u'58 m2 each plot' u'120 m2 each plot'
 u'25 m2 each plot' u'90 m2 each plot' u'150 m2 each plot'
 u'17 m2 each plot' u'1750 m2 each plot' u'1400 m2 each plot'
 u'20 m x 8 m each subplot' u'9 m x 20 m each sub-plot'
 u'10 m x 25 m each plot' u'22 m x 22 m' u'36 m x 29 m' u'20 m x 30 m'
 u'20 m x 25 m' u'8 m x 13 m' u'84 m2' u'31.2 m2 each plot' u'33 ha'
 u'15 ha' u'2 ha' u'11 ha' u'0.5 ha' u'0.75 ha' u'6000 m2' u'3800 m2'
 u'4000 m2' u'7 m x 7 m each plot' u'5 m x 5 m each plot'
 u'3.6 m x 3.6 m each plot' u'6 m x 6 m each plot' u'20 m x 20 m each plot'
 u'40 m x 40 m each plot' u'2500 m2 each plot' u'1 m x 1 m each plot'
 u'2.74 m x 3.96 m each plot' u'7.3 m x9.1 m each plot' u'158 m2 each plot'
 u'642 m2' u'4 m x 5 m each plot' u'20 m x 50 m each plot'
 u'13 m x 6 m each plot' u'12 m x 18 m each plot' u'6.75 m x 7 m each plot'
 u'75 m2 each plot' u'23 m x 28 m each plot' u'640 m2'
 u'ranging from 27 m x 27 m to 36 m x 27 m' u'15 m x 30 m each plot'
 u'325 m2 for measurement plots' u'90 ha' u'210 m x 210 m each plot'
 u'380 m2 each plot, internal measurement 144 m2' u'0.17 ha each plot'
 u'54 m x 75 m each plot' u'13 m x 20 m each plot' u'25 m x 36 m each plot'
 u'50 m x 50 m each sampling site' u'9 m x 9 m each subplot'
 u'ranging from  0.04 ha to 0.2 ha each plot' u'22 m x 22 m each plot'
 u'0.2 ha each plot' u'16 m x 16 m each plot' u'6 m x 10.5 m each plot'
 u'50 m x 40 m each plot' u'80000 ha' u'0.01 ha or 0.02 ha' u'0.1 ha'
 u'0.06 ha' u'30 m2 each plot' u'10000 m2 each plot' u'124.8 m2 each plot'
 u'12.1 m  x 13.2 m each plot' u'4 ha, about 47 m2 each plot'
 u'0.25 ha each sampling plot' u'75 ha' u'3 m x 9 m each plot'
 u'24 m2 each plot' u'7.6 m x 1.5 m each plot' u'3 m x 9.1 m each plot'
 u'2.43 ha' u'0.4 ha' u'0.405 ha' u'14 m x 8.4 m each plot'
 u'4 m x 6 m each plot' u'2430 m2' u'396 m2' u'440 m2' u'4050 m2'
 u'2320 m2' u'5203 m2' u'7.2 m x 7.2 m each plot' u'3 m x 3 m each plot'
 u'228 m2 each plot' u'189 m2 each plot' u'2 m x 7 m each plot'
 u'total surface 0.55 ha' u'total surface 0.62 ha'
 u'6.1 m x 6.1 m each plot' u'450 m2' u'450 m2 total area, 25 m2 each plot'
 u'1.5 m x 6.1 m each plot' u'plantation size 0.04 ha'
 u'plantation size 0.19 ha' u'plantation size 0.004 ha'
 u'plantation size 0.015 ha' u'plantation size 0.059 ha'
 u'12 m x 30 m each plot' u'2 m x 3 m each plot' u'1.5 ha total area'
 u'2.7 ha total area, 17.25 m2 each sampling plot' u'220.5 m2 each plot'
 u'6 m x 7 m each subplot' u'4.5 m x 6 m each plot'
 u'5.2 m x 9.1 m each plot' u'6.67 m x 3.75 m each plot'
```

```
 u'3.2 m x 4.8 m each plot' u'9 m2 of harvesting area each plot'
 u'8 m x 3 m' u'2 m x 5 m each plot' u'7 m x 6 m each plot'
 u'7 m x 9 m each plot' u'ranging from 12 m2 to 30 m2 each plot'
 u'total area 4.1 ha, 624 m2 each plot' u'3.6 m x 7 m each plot'
 u'6 m x 7 m each plot' u'6 m x 5 m each plot' u'4 m x 10 m each plot'
 u'5 m x 10 m each plot' u'60 m x 24 m each plot'
 u'2400 m2 examined area, 2 m x 2 m each sampling plot'
 u'4500 m2 examined area, 2 m x 2 m each sampling plot'
 u'180 m2 examined area, 2 m x 2 m each sampling plot'
 u'2430 m2 examined area, 2 m x 2 m each sampling plot' u'667 m2'
 u'50 m x 20 m each sampling plot' u'450 m2 each plot'
 u'20 m x 30 m each sampling plot'
 u'ranging from 0.01 to 0.04 ha each plot'
 u'30 m x 20 m each sampling plot' u'3600 m2'
 u'about 10 ha total area, 30 m x 20 m each plot'
 u'20 m x 20 m each sampling plot'
 u'6.67 ha total area, 100 m2 each sampling plot'
 u'400 m2 each sampling plot' u'6.67 ha total area'
 u'40 m x 20 m each sampling plot' u'25 m x 25 m each plot'
 u'5 ha total area, ranging from 2300 to 4400 m2 each plot'
 u'4 m x 4 m each plot' u'1.5 m x 2 m each plot'
 u'21.6 ha, 25 m x 25 m each sampling plot' u'50 m2 each plot'
 u'0.1 ha each sampling plot'
 u' 2997.776 ha total area, 100 m x 100 m each sampling plot'
 u'7.3 ha total area, ' u'25 m x 40 m each sampling plot'
 u'21 m2 each plot' u'30 ha' u'22.5 m2 each plot' u'10 m2 each plot'
 u'24 m x 24 m' u'30 m x 24 m' u'204 m x 106 m' u'67000 ha' u'4 ha'
 u'50 m x 50 m each sampling plot' u'220 ha total area'
 u'667 m2 each sampling plot' u'16 m x 16 m or 8 m x 8 m each plot'
 u'4.6 m x 2.1 m each plot']
```

### 3.3.2 Crop_type, Species, Detailed_species_information

```
In [55]: print '-'*80
         print 'Crop_type, unique values:\n', np.sort(df1.Crop_type.unique())
         print '-'*80
         print 'Species, unique values:\n', np.sort(df1.Species.unique())
```

```
--------------------------------------------------------------------------------
Crop_type, unique values:
[nan u'Acacia' u'Albizia' u'Alder' u'Alfalfa' u'Alfalfa+Sorghum'
 u'Alfalfa-bromegrass' u'Arundo' u'Bermudagrass' u'Big Bluestem'
 u'Big bluestem' u'Bromegrass' u'Casuarina' u'Casuarina+Eucalyptus'
 u'Casuarina+Leucaena' u'Cocksfoot grass' u'Common reed' u'Cordgrass'
 u'Cynara' u'Dalbergia' u'Eastern gamagrass' u'Elephantgrass' u'Energycane'
 u'Enterolobium' u'Erianthus' u'Eucalyptus' u'Eucalyptus+Acacia'
 u'Eucalyptus+Albizia' u'Eucalyptus+Facaltaria' u'Eucalyptus+Leucaena'
```

```
 u'Facaltaria' u'Fescue' u'Fiber sorghum' u'Flaccidgrass' u'Flatpea'
 u'Forage brassica' u'Giant reed' u'Gliricidia' u'Hemp' u'Indian grass'
 u'Johnsongrass' u'Kale' u'Leucaena' u'Lovegrass' u'Maize' u'Meadow'
 u'Milkvetch' u'Miscanthus' u'Napiergrass' u'Panic grass' u'Paraserianthes'
 u'Pinus' u'Poplar' u'Reed canary grass' u'Reed canarygrass' u'Rye'
 u'Sandreed' u'Silverreed' u'Sorghum' u'Sorghum-sudangrass' u'Sudangrass'
 u'Sugercane' u'Sweetcane' u'Switchgrass' u'Switchgrass+Milkvetch'
 u'Tall fescue' u'Timothy-redtop-red clover' u'Topinambur' u'Treiticale'
 u'Triticale' u'Virginia fanpetals' u'Wheatgrass' u'Willow'
 u'big cordgrass' u'elephantgrass' u'energycane' u'galingale' u'giant reed'
 u'grass mixture' u'guinea grass' u'napier grass' u'napier hybrid'
 u'prairie cordgrass']
--------------------------------------------------------------------------------
Species, unique values:
[nan
 u'(Populus deltoides x Populus deltoides) x (Populus nigra x Populus nigra)'
 u'(Populus deltoides x Populus deltoides) x Populus nigra'
 u'(Populus trichocarpa x Populus deltoides) x (Populus trichocarpa x Populus deltoides)'
 u'(Populus x generosa) x Populus nigra'
 u'(Populus x generosa) x Populus trichocarpa'
 u'(Salix babylonica x Salix alba) x Salix matsudana'
 u'(Salix matsudana x Chosenia arbutifolia) x Salix matsudana'
 u'(Salix schwerinii x Salix viminalis) x Salix viminalis'
 u'(Salix viminalis x  Salix viminalis) x Salix viminalis'
 u'(Salix viminalis x Salix eriocephala) x Salix viminalis'
 u'(Salix viminalis x Salix triandra) x Salix miyabeana'
 u'Acacia auriculiformis' u'Acacia hybrid' u'Acacia mangium'
 u'Acacia mearnsii' u'Agropyron desertorum' u'Albizia falcataria '
 u'Albizia procera' u'Alnus rubra' u'Andropogon gerardii'
 u'Andropogon gerardii ' u'Arundo donax' u'Astragalus adsurgens'
 u'Brassica napus' u'Brassica oleracea' u'Bromus inermis'
 u'Calamovilfa longifolia' u'Cannabis sativa' u'Casuarina equisetifolia'
 u'Casuarina equisetifolia + Eucalyptus x robusta'
 u'Casuarina equisetifolia + Leucaena leucocephala' u'Cynara cardunculus'
 u'Cynodon dactylon' u'Cyperus longus' u'Dactylis glomerata'
 u'Dalbergia sissoo' u'Enterolobium cyclocarpum' u'Eragrostis curvula'
 u'Erianthus spp.' u'Eucalyptus Lezhou' u'Eucalyptus amygdalina'
 u'Eucalyptus botryoides' u'Eucalyptus botryoides x saligna'
 u'Eucalyptus brookerana' u'Eucalyptus camaldulensis'
 u'Eucalyptus coccifera' u'Eucalyptus cordata' u'Eucalyptus diversicolor'
 u'Eucalyptus elata' u'Eucalyptus eucoxylon' u'Eucalyptus globoidea'
 u'Eucalyptus globulus' u'Eucalyptus globulus + Acacia mearnsii'
 u'Eucalyptus grandis' u'Eucalyptus grandis + Acacia mangium'
 u'Eucalyptus grandis x Eucalyptus tereticornis'
 u'Eucalyptus grandis x Eucalyptus urophylla'
 u'Eucalyptus grandis x urophglla' u'Eucalyptus grandis x urophylla'
 u'Eucalyptus leizhou' u'Eucalyptus macarthurii' u'Eucalyptus maideni'
 u'Eucalyptus nitens' u'Eucalyptus nitida' u'Eucalyptus obliqua'
```

```
u'Eucalyptus ovata' u'Eucalyptus pellita' u'Eucalyptus pseudoglobulus'
u'Eucalyptus pulchella' u'Eucalyptus regnans' u'Eucalyptus robusta Smith'
u'Eucalyptus rodwayii' u'Eucalyptus saligna'
u'Eucalyptus saligna + Albizia falcataria'
u'Eucalyptus saligna + Facaltaria moluccana' u'Eucalyptus smithii'
u'Eucalyptus tereticornis' u'Eucalyptus urnigera' u'Eucalyptus urophylla'
u'Eucalyptus urophylla + Leucaena leucocephala'
u'Eucalyptus urophylla x Eucalyptus grandis'
u'Eucalyptus urophylla x Eucalyptus tereticornis' u'Eucalyptus viminalis'
u'Eucalyptus x robusta' u'Eucalyptus x robusta + Leucaena leucocephala '
u'Facaltaria moluccana' u'Festuca arundinacea' u'Gliricidia sepium'
u'Gmelina arborea' u'Helianthus tuberosus' u'Lathyrus sylvestris'
u'Leucaena  leucocephala x Leucaena diversifolia' u'Leucaena diversifolia'
u'Leucaena leucocephala' u'Medicago sativa'
u'Medicago sativa + Sorghum bicolor' u'Megathyrsus maximus'
u'Miscanthus floridulus' u'Miscanthus giganteus'
u'Miscanthus lutarioriparius' u'Miscanthus sacchariflorus'
u'Miscanthus sacchariflorus x Miscanthus sinensis' u'Miscanthus sinensis'
u'Miscanthus sinensis x Miscanthus sacchariflorus'
u'Miscanthus sinensis x Miscanthus sinensis' u'Miscanthus x giganteus'
u'Miscanthus x giganteus and Miscanthus sinensis' u'Panicum amarum'
u'Panicum pratense' u'Panicum virgatum' u'Panicum virgatum L.'
u'Panicum virgatum+ Astragalus adsurgens' u'Paraserianthes falcataria'
u'Pascopyrum smithii' u'Pascopyrum smithii ' u'Pennisetum flaccidum'
u'Pennisetum purpureum' u'Phalaris arundinacea'
u'Phleum pratense, Agrostis gigantea, Trifolium pratense'
u'Phragmites australis' u'Pinus radiata' u'Polupus tomentosa'
u'Populus Beijingensis ' u'Populus alba'
u'Populus alba and Populus russkii' u'Populus alba x Populus alba'
u'Populus alba x Populus bero' u'Populus balsamifera'
u'Populus balsamifera x Populus laurifolia'
u'Populus balsamifera x Populus tremula'
u'Populus balsamifera x Populus trichocarpa'
u'Populus balsamifera x Populus tristis' u'Populus canadaensis'
u'Populus canadaensis ' u'Populus deltoides'
u'Populus deltoides x (Populus x euramericana)'
u'Populus deltoides x Populus deltoides'
u'Populus deltoides x Populus nigra'
u'Populus deltoides x Populus trichocarpa' u'Populus euphratica'
u'Populus euramericana x Populus deltoides' u'Populus euramevicana'
u'Populus eurasia x yunnanensis'
u'Populus maximowiczii x Populus berolinensis'
u'Populus maximowiczii x Populus deltoides'
u'Populus maximowiczii x Populus nigra'
u'Populus maximowiczii x Populus trichocarpa' u'Populus nigra'
u'Populus nigra x Populus maximowiczii' u'Populus russkii'
u'Populus schneideri' u'Populus simonii'
u'Populus simonii x Populus pyramidalis' u'Populus szechuanica'
```

```
u'Populus tomentosa' u'Populus trchocarpa'
u'Populus trchocarpa x Populus deltoides'
u'Populus tremula x Populus tremula'
u'Populus tremula x Populus tremuloides'
u'Populus tremula x Pupulus tremuloides'
u'Populus tremuloides x Populus tremula'
u'Populus tremuloides x Populus tremuloides' u'Populus trichocarpa'
u'Populus trichocarpa ' u'Populus trichocarpa x Populus balsamifera'
u'Populus trichocarpa x Populus deltoides'
u'Populus trichocarpa x Populus koreana'
u'Populus trichocarpa x Populus nigra / Populus trichocarpa x Populus angustifolia'
u'Populus trichocarpa x Populus trichocarpa'
u'Populus trichocarpa, Populus deltoides' u'Populus tristis'
u'Populus x canadensis' u'Populus x euramericana'
u'Populus x euramericana ' u'Populus x rasumowskyana' u'Populus x xiaohei'
u'Populus x xiaozhuanica ' u'Saccharum arundinaceum' u'Saccharum hybrid'
u'Saccharum officinarum ' u'Saccharum spp.' u'Salix Jiangsuensis'
u'Salix alba' u'Salix alba and Salix matsudana' u'Salix alba x Salix alba'
u'Salix alberti x Salix x leucopithecia' u'Salix americana'
u'Salix babylonica x Salix humboldtiana'
u'Salix burjactica x Salix dasyclados'
u'Salix burjactica x Salix viminalis' u'Salix cheilophila'
u'Salix chelophila' u'Salix dasyclados'
u'Salix dasyclados / Salix burjactica'
u'Salix dasyclados x Salix dasyclados' u'Salix dasyclados x purpurea'
u'Salix discolor' u'Salix erio x Salix erio'
u'Salix erio x Salix interior' u'Salix erio x Salix petiolaris'
u'Salix eriocephala' u'Salix eriocephala x Salix interior'
u'Salix fragilis' u'Salix hybrid' u'Salix integra'
u'Salix interior x Salix eriocephala' u'Salix jessoensis'
u'Salix koriyanagi x Salix purpurea' u'Salix matsudana'
u'Salix matsudana x (Salix matsudana x Salix alba), Salix alba '
u'Salix matsudana x Salix alba' u'Salix matsudana x alba'
u'Salix miyabeana' u'Salix psammophila' u'Salix purpurea'
u'Salix purpurea x Salix miyabeana' u'Salix redheriana x Salix dasyclados'
u'Salix sachalinensis' u'Salix schwerini'
u'Salix schwerinii x Salix viminalis' u'Salix spp.'
u'Salix triandra x Salix viminalis' u'Salix viminalis'
u'Salix viminalis x (Salix schwerinii x Salix viminalis)'
u'Salix viminalis x Salix burjactica' u'Salix viminalis x Salix miyabeana'
u'Salix viminalis x Salix schwerinii' u'Salix viminalis x Salix viminalis'
u'Salix x dasyclados' u'Salix x jiangsuensis' u'Salix x rubra'
u'Secale cereale' u'Secale montanum' u'Sida hermaphrodita'
u'Sorghastrum nutans' u'Sorghum bhalepense ' u'Sorghum bicolor'
u'Sorghum bicolor x Salix sudanense' u'Sorghum sudanensis'
u'Spartina cynosuroides' u'Spartina pectinata' u'Thinopyrum intermedium'
u'Thinopyrum intermedium, Thinopyrum ponticum, Medicago sativa, Melilotus officinalis'
u'Triarrhena sacchariflora' u'Tripsacum dactyloides'
```

```
 u'Triticosecale Wittmack' u'Zea mays' u'mixed species'
 u'x Triticosecale schlanstedtense']
```

```
In [56]: print '-'*80
         print 'Detailed_species_information, unique values:\n',
         np.sort(df1.Detailed_species_information.unique())
```

```
--------------------------------------------------------------------------------
Detailed_species_information, unique values:
```

```
Out[56]: array([nan, u' P. nigra, Wolterson',
                u'(Jorunn x Salix eriocephala) x Bjorn, Sherwood',
                u"(Populus deltoides Bartr. cv. 'Shanhaiguanensis' x Populus deltoides cl. 'Ha
                u'(Populus x generosa) x Populus nigra, AF6',
                u'(Populus x generosa) x Populus nigra, Monviso',
                u'(Populus x generosa) x Populus trichocarpa, AF8',
                u'(SW930812 Jorunn x Bjorn) x Quest, Resolution',
                u'(Salix babylonica x Salix alba) x Salix matsudana f. lobatoglandulosa, SE03-
                u'(Salix matsudana x Chosenia arbutifolia) x Salix matsudana f. lobatoglandulo
                u'(Salix matsudana x Chosenia arbutifolia) x Salix matsudana f. lobatoglandulo
                u'(Salix schwerinii x Salix viminalis) x Salix viminalis, Trodis',
                u'(Salix viminalis Bowles Hybrid x Salix triandra Dark Newkind) x Salix miyabe
                u'(Tora x Jorr) x Ivar, Karin', u'A. mangium', u'A. mearnsii',
                u'Acacia auriculiformis A. Cunn. ex Benth',
                u'Acacia hybrid, clone B-10', u'Acacia hybrid, clone B-5',
                u'Acacia hybrid, clone H-10', u'Acacia hybrid, clone H-4',
                u'Acacia hybrid, clone H12', u'Acacia hybrid, clone HD-12',
                u'Acacia hybrid, clone HD-16', u'Acacia hybrid, clone HD20',
                u'Acacia hybrid, clone K-23', u'Acacia hybrid, clone K-40',
                u'Acacia hybrid, clone K26', u'Acacia hybrid, clone Normal',
                u'Acacia mearnsii',
                u'Agropyron desertorum (Fisch. ex Link), Crested wheatgrass',
                u'Albizia falcataria (L.) = Fosberg Paraserianthes facaltaria (L.) Nielson',
                u'Albizia falcataria (L.) Fosberg Paraserianthes facaltaria (L.) Nielson',
                u'Alnus rubra Bong., red alder',
                u'Andropogon gerardii Vitman var. gerardii',
                u'Andropogon gerardii Vitman, Bison, Bison',
                u'Andropogon gerardii Vitman, Niagara, NBB', u'Arundo donax L.',
                u'Arundo donax L. cv. Wild ecotype', u'Astragalus adsurgens Pall.',
                u'Banagrass, NG1', u'Brassica napus', u'Brassica oleracea',
                u'Bromus inermis Leyss., Bromegrass',
                u'Calamovilfa longifolia (Hook.) Scibn., 9004944, Ca44',
                u'Calamovilfa longifolia (Hook.) Scibn., 9004959, Ca59',
                u'Calamovilfa longifolia (Hook.) Scibn., ND95',
                u'Calamovilfa longifolia (Hook.) Scibn., PI477011, PI4',
                u'Calamovilfa longifolia (Hook.) Scibn., Pronghorn, Pron',
```

```
u'Cannabis sativa L.', u'Casuarina equisetifolia',
u'Casuarina equisetifolia 50: Eucalyptus x robusta 50',
u'Casuarina equisetifolia 50: Leucaena leucocephala 50',
u'Cynara cardunculus L. var. altilis D.C., Cardo gigante inerme',
u'Cyperus longus L.', u'D. sissoo Roxb', u'Dactylis glomerata L.',
u'E. globulus (Labill.)',
u'E. grandis + E. cyclocarpum, mixed forest',
u'E. grandis + Leucaena hybrid KX3, mixed forest',
u'E. grandis + P. falcataria, mixed forest',
u"Eragrostis curvula cv 'Common'", u'Erianthus spp.',
u'Eucalyptus 12 ABL', u'Eucalyptus Lezhou No. 1',
u'Eucalyptus botryoides, Clone 3734',
u'Eucalyptus camaldulensis Dehn.',
u'Eucalyptus camaldulensis Dehnh',
u'Eucalyptus camaldulensis Dehnh, Clone 3735',
u'Eucalyptus camaldulensis Dehnh. (Red River gum)',
u'Eucalyptus diversicolor F. Muell.', u'Eucalyptus globulus Labill',
u'Eucalyptus globulus Labill.',
u'Eucalyptus globulus ssp. globulus',
u'Eucalyptus globulus ssp. pseudoglobulus',
u'Eucalyptus globulus ssp. pseudoglobulus (Naudin ex Maiden) Kirkpatr.',
u'Eucalyptus globulus ssp. pseudoglobulus 25% + Acacia mearnsii 75%',
u'Eucalyptus globulus ssp. pseudoglobulus 50% + Acacia mearnsii 50%',
u'Eucalyptus globulus ssp. pseudoglobulus 75% + Acacia mearnsii 25%',
u'Eucalyptus grandis',
u'Eucalyptus grandis (W. Hill ex Maiden) x Eucalyptus urophylla (SalixT. Blake)
u'Eucalyptus grandis 100: A. mangium 100',
u'Eucalyptus grandis 100: A. mangium 25 ',
u'Eucalyptus grandis 100: A. mangium 50',
u'Eucalyptus grandis 50: A. mangium 50',
u'Eucalyptus grandis Hill ex Maiden',
u'Eucalyptus grandis Hill ex Maiden (flooded gum)',
u'Eucalyptus grandis W. Hill ex Maiden',
u'Eucalyptus grandis W. Hill ex. Maiden x Eucalyptus urophylla S.T. Blake',
u'Eucalyptus grandis x Eucalyptus tereticornis, DH201-2',
u'Eucalyptus grandis x Eucalyptus tereticornis, clone DH201',
u'Eucalyptus grandis, Clone 3736', u'Eucalyptus leizhou, SH7',
u'Eucalyptus macarthurii, Clone 3737',
u'Eucalyptus nitens, Clone 3738', u'Eucalyptus ovata, Clone 3739',
u'Eucalyptus pseudoglobulus, Clone 3740', u'Eucalyptus saligna',
u'Eucalyptus saligna 25% + Facaltaria moluccana 75%',
u'Eucalyptus saligna 34% + Albizia falcataria 66%',
u'Eucalyptus saligna 50% + Albizia falcataria 50%',
u'Eucalyptus saligna 50% + Facaltaria moluccana 50%',
u'Eucalyptus saligna 66% + Albizia falcataria 34%',
u'Eucalyptus saligna 67% + Facaltaria moluccana 33%',
u'Eucalyptus saligna 75% + Albizia falcataria 25%',
u'Eucalyptus saligna 75% + Facaltaria moluccana 25%',
```

u'Eucalyptus saligna 89% + Albizia falcataria 11%',
u'Eucalyptus saligna 89% + Facaltaria moluccana 11%',
u'Eucalyptus saligna Sm.', u'Eucalyptus saligna, Clone 3741',
u'Eucalyptus urophylla',
u'Eucalyptus urophylla + Leucaena leucocephala K72',
u'Eucalyptus urophylla + Leucaena leucocephala K8',
u'Eucalyptus urophylla S. T. Blake',
u'Eucalyptus urophylla S. T. Blake x Eucalyptus tereticornis Smith',
u'Eucalyptus urophylla x Eucalyptus grandis, DH32-22',
u'Eucalyptus urophylla x Eucalyptus grandis, EC34',
u'Eucalyptus urophylla x Eucalyptus grandis, GL4',
u'Eucalyptus urophylla x Eucalyptus grandis, GL9',
u'Eucalyptus urophylla x Eucalyptus tereticornis, LH1',
u'Eucalyptus urophylla x Eucalyptus tereticornis, LH5',
u'Eucalyptus urophylla x Eucalyptus tereticornis, M1',
u'Eucalyptus urophylla x Eucalyptus tereticornis, UC184-1',
u'Eucalyptus viminalis, Clone 3673',
u'Eucalyptus viminalis, Clone 3674',
u'Eucalyptus viminalis, Clone 3678',
u'Eucalyptus viminalis, Clone 3679',
u'Eucalyptus viminalis, Clone 3680',
u'Eucalyptus viminalis, Clone 3683',
u'Eucalyptus viminalis, Clone 3685',
u'Eucalyptus viminalis, Clone 3686',
u'Eucalyptus viminalis, Clone 3702',
u'Eucalyptus viminalis, Clone 3704', u'Eucalyptus x robusta',
u'Eucalyptus x robusta 50: Leucaena leucocephala 50',
u'Facaltaria moluccana',
u'Facaltaria moluccana(Miquel) Barneby &  Grimes (=Albizia Facaltaria (L.) Fos
u"Festuca arundinacea 'dulcia', Festuca arundinacea 'dulcia'  'Noria'",
u"Festuca arundinacea cv 'Forager'",
u'Gliricidia sepium (Jacq.) Steud', u'Helenvale provenance',
u'Helianthus tuberosus L., Parlow', u'Jorunn x Bjorn, Sven',
u'K06, GG3', u'Lathyrus sylvestris L.',
u'Leucaena  leucocephala x Leucaena diversifolia Benth. KX3',
u'Leucaena diversifolia (Schlecht.) Benth.',
u'Leucaena leucocephala', u'Leucaena leucocephala, K72',
u'Leucaena leucocephala, K8', u"M. sinensis 'Giganteus'",
u'M. sinensis Hybrid, hybrid of M. sacchariflorus x M. sinensis',
u'M. sinensis Hybrid, hybrid of two M. sinensis',
u'M. sinensis Hybrid, hybrid slected in a M. sinensis population',
u'MG04, GG4', u"Medicago sativa 'Alpha' , Medicago sativa 'Orca'",
u'Medicago sativa L., Brome-alfalfa',
u'Medicago sativa, Bromus inermis',
u'Medicago sativa, cv. Hamedani',
u'Medicago sativa, cv. Hamedani, 25% + Sorghum bicolor, cv. Kimia, 75%',
u'Medicago sativa, cv. Hamedani, 50% + Sorghum bicolor, cv. Kimia, 50%',
u'Medicago sativa, cv. Hamedani, 75% + Sorghum bicolor, cv. Kimia, 25%',

```
u'Merkeron, NG3', u'Miscanthus floridulus, Flo, 3x',
u'Miscanthus sacchariflorus',
u'Miscanthus sacchariflorus (Maxim.) Franch.',
u'Miscanthus sacchariflorus Maxim. Benth.',
u'Miscanthus sacchariflorus x Miscanthus sinensis, 10',
u'Miscanthus sacchariflorus x Miscanthus sinensis, 8',
u'Miscanthus sacchariflorus, H10, 4x',
u'Miscanthus sacchariflorus, Sac, 2x', u'Miscanthus sinensis',
u"Miscanthus sinensis 'Giganteus'",
u"Miscanthus sinensis 'Giganteus', clone Hornum",
u'Miscanthus sinensis (2x), MS/5',
u'Miscanthus sinensis (2x), MS/6',
u"Miscanthus sinensis cv. 'Giganteus'",
u'Miscanthus sinensis cv. Giganteus',
u'Miscanthus sinensis hybrids',
u'Miscanthus sinensis hybrids, Sin-H9',
u'Miscanthus sinensis triploid, 6',
u'Miscanthus sinensis x Miscanthus sacchariflorus, MS/3',
u'Miscanthus sinensis x Miscanthus sacchariflorus, MS/4',
u'Miscanthus sinensis x Miscanthus sinensis, 7',
u'Miscanthus sinensis, Aug, 2x', u'Miscanthus sinensis, Fer, 2x',
u'Miscanthus sinensis, Fla, 2x', u'Miscanthus sinensis, GoD, 4x',
u'Miscanthus sinensis, Gol, 4x', u'Miscanthus sinensis, Goliath',
u'Miscanthus sinensis, Grz, 2x', u'Miscanthus sinensis, H6, 3x',
u'Miscanthus sinensis, Her, 2x', u'Miscanthus sinensis, Mal, 2x',
u'Miscanthus sinensis, Punk, 2x',
u'Miscanthus sinensis, Purple, NG2, 2x',
u'Miscanthus sinensis, Rot, 2x', u'Miscanthus sinensis, Sil, 2x',
u'Miscanthus sinensis, Sin-13', u'Miscanthus sinensis, Sin-15',
u'Miscanthus sinensis, Str, 2x', u'Miscanthus sinensis, Yak, 2x',
u'Miscanthus sinensis, pure, 11', u'Miscanthus sinensis, pure, 12',
u'Miscanthus sinensis, pure, 13', u'Miscanthus sinensis, pure, 14',
u'Miscanthus sinensis, pure, 15', u'Miscanthus x giganteus',
u'Miscanthus x giganteus Greef &Deuter ex Hodkinson & Renvoize',
u'Miscanthus x giganteus Greef et Deu ex. Hodkinson et Renvoize, "Illinois" cl
u'Miscanthus x giganteus Greef et Deu.',
u'Miscanthus x giganteus Greef et Deuter',
u'Miscanthus x giganteus Greef et Deuter ex Hodkinson et Renvoize',
u'Miscanthus x giganteus L.', u'Miscanthus x giganteus, Gig-1',
u'Miscanthus x giganteus, Gig-2',
u'Miscanthus x giganteus, GigB, 3x',
u'Miscanthus x giganteus, GigD, 4x',
u'Miscanthus x giganteus, H8, 2x',
u'Miscanthus x giganteus, triploid (3x) hybrid, MG/1',
u'Miscanthus x giganteus, triploid (3x) hybrid, MG/2', u'OG03, GG1',
u'OG05, GG2', u'P. Beijingensis W.H. Hsu', u'P. alba L.',
u'P. alba x P. bero linensis', u'P. canadaensis ',
u'P. canadaensis Moench Cv.', u'P. deltoides x P. nigra, Gaver',
```

u'P. deltoides x P. nigra, Gibercq',
u'P. deltoides x P. nigra, Primo',
u'P. deltoides x P. trichocarpa, IBW1 DxT',
u'P. deltoides x P. trichocarpa, IBW2 DxT',
u'P. deltoides x P. trichocarpa, IBW3 DxT', u'P. deltoides, 80-020',
u'P. deltoides, 84-078', u'P. deltoides, 85-037',
u'P. deltoides, Baldo', u'P. deltoides, Dvina',
u'P. deltoides, Lambro', u'P. deltoides, Lena',
u'P. deltoides, Lux', u'P. deltoides, Oglio', u'P. euphratica oliv',
u'P. euramericana cv. 1-72/58', u'P. maximowiczii x P. trichocarpa',
u'P. nigra clone 107', u'P. nigra clone 113', u'P. nigra clone 202',
u'P. nigra clone 206', u'P. nigra clone 210', u'P. nigra clone 301',
u'P. nigra clone 302', u'P. nigra clone 309', u'P. nigra clone 311',
u'P. nigra var. thevestina', u'P. schneideri var. tibetica',
u'P. szechuanica var. tibetica Schneid', u'P. tomentosa carr',
u'P. trchocarpa (Trichobel)',
u'P. trchocarpa x P. deltoides (Beaupre)',
u'P. trchocarpa x P. deltoides (Boelare)',
u'P. trichoarpa x P. deltoides, 11-11',
u'P. trichoarpa x P. deltoides, 47-174',
u'P. trichoarpa x P. deltoides, 49-177',
u'P. trichoarpa x P. deltoides, H-11', u'P. trichocarpa CL',
u'P. trichocarpa Columbia River', u'P. trichocarpa Fritzi Pauley',
u'P. trichocarpa Trichobel',
u'P. trichocarpa and P. deltoides, pure or hybrid',
u'P. trichocarpa x P. balsamifera, Balsam Spire',
u'P. trichocarpa x P. deltoides, Beaupre',
u'P. trichocarpa x P. deltoides, Boelare',
u'P. trichocarpa x P. deltoides, Hazendans',
u'P. trichocarpa x P. deltoides, Hoogvorst',
u'P. trichocarpa x P. deltoides, Raspalje',
u'P. trichocarpa x P. deltoides, Unal',
u'P. trichocarpa, pure species', u'P. x canadensis, 83.039.009',
u'P. x canadensis, 83.039.018', u'P. x canadensis, 83.141.020',
u'P. x canadensis, BL-Costanzo', u'P. x canadensis, Cima',
u'P. x canadensis, I-214', u'P. x canadensis, Luisa Avanzo',
u'P. x canadensis, Orion', u'P. x euramericana Guinier',
u'Panicum amarum A.S. Hitchc. & Chase, Atlantic PG',
u'Panicum pratense', u'Panicum virgatum ',
u"Panicum virgatum 'Kanlow'",
u'Panicum virgatum : Astragalus adsurgens Pall. = 1:1',
u'Panicum virgatum : Astragalus adsurgens Pall. = 2:1',
u'Panicum virgatum L.', u'Panicum virgatum L. "Kanlow"',
u'Panicum virgatum L. cv. Cave-in-Rock',
u'Panicum virgatum L., Alamo', u'Panicum virgatum L., Blackwell',
u'Panicum virgatum L., Cave-in-Rock',
u'Panicum virgatum L., Cave-in-Rock (CIR)',
u'Panicum virgatum L., Cave-in-Rock (CIR), Pathfinder (PF), Sunburst (SB)',

```
u'Panicum virgatum L., Cave-in-Rock, CIR',
u'Panicum virgatum L., Cave-in-Rock, Shelter, Alamo, Kanlow, NC-1, NC-2',
u'Panicum virgatum L., Cave-in-rock',
u'Panicum virgatum L., Dacotah', u'Panicum virgatum L., Dakota',
u'Panicum virgatum L., Dakota, DK',
u'Panicum virgatum L., Forestberg',
u'Panicum virgatum L., Forestburg',
u'Panicum virgatum L., Illinois', u'Panicum virgatum L., Kanlow',
u'Panicum virgatum L., Kansas Native', u'Panicum virgatum L., NC-1',
u'Panicum virgatum L., NC-2', u'Panicum virgatum L., ND3743',
u'Panicum virgatum L., ND3743, ND', u'Panicum virgatum L., NJ-50',
u'Panicum virgatum L., Nebraska 28',
u'Panicum virgatum L., Nebraska synthetic, NEB',
u'Panicum virgatum L., New Jersey 50',
u'Panicum virgatum L., New Jersey 50, NJ50',
u'Panicum virgatum L., Ottawa1, OTT1',
u'Panicum virgatum L., Ottawa2 OTT2',
u'Panicum virgatum L., Ottawa3, OTT3',
u'Panicum virgatum L., Ottawa4, OTT4',
u'Panicum virgatum L., Pathfinder',
u'Panicum virgatum L., Pathfinder (PF)',
u'Panicum virgatum L., Pathfinder, PF',
u'Panicum virgatum L., Shelter',
u'Panicum virgatum L., Shelter, SH', u'Panicum virgatum L., Summer',
u'Panicum virgatum L., Sunburst',
u'Panicum virgatum L., Sunburst (SB)',
u'Panicum virgatum L., Trailblazer',
u'Panicum virgatum L., Trailblazer, TL',
u'Panicum virgatum L., cv Cave-in-Rock',
u"Panicum virgatum cv 'Cave-in-Rock'",
u'Panicum virgatum cv. Alamo', u'Panicum virgatum, Cave in Rock',
u'Panicum virgatum, Kanlow',
u'Pascopyrum smithii (Rydb.) A. Love, I & western weatgrass',
u'Pearl millet x dwarf, PMxD', u'Pennisetum purpureum Schum.',
u'Pennisetum purpureum Schum. cv. "Merkeron"',
u'Pennisetum purpureum Schum. cv. Merkeron',
u'Pennisetum purpureum, Merkeron', u'Pennisetum purpureum, UF1',
u'Petford provenance', u'Phalaris arundinacea L.',
u'Phalaris arundinacea, Bamse', u'Phalaris arundinacea, Palaton',
u'Phleum pratense, Agrostis gigantea L., Trifolium pratense',
u'Phragmites australis', u'Populus alba L., 93.088.006',
u'Populus alba L., 93.088.015', u'Populus alba L., 93.088.047',
u'Populus alba L., 93.088.091', u'Populus alba L., 93.088.095',
u'Populus alba L., 93.088.133', u'Populus alba L., 93.088.145',
u'Populus alba L., 93.088.202', u'Populus alba L., 93.088.232',
u'Populus alba L., DI-102', u'Populus alba L., GA-107-D',
u'Populus alba L., PA078', u'Populus alba L., PA079',
u'Populus alba L., PA085', u'Populus alba L., PA089',
```

```
u'Populus alba L., PI93.006', u'Populus alba L., PI93.007',
u'Populus alba L., PI93.008', u'Populus alba L., PI93.022',
u'Populus alba L., VILLAFRANCA', u'Populus alba var. pyramidalis ',
u'Populus alba var. pyramidalis and Populus russkii',
u"Populus balsamifera L. x Populus tristis Frisch,  'Tristis #1'",
u'Populus balsamifera var. Michauxii (Herry) x Populus trichocarpa var. Hastata
u'Populus balsamifera x Populus tremula cf. balsamifera, P-524',
u'Populus balsamifera, 910, 51', u"Populus deltoides 'G3'",
u'Populus deltoides 1112', u'Populus deltoides 2059',
u'Populus deltoides 26C6R51',
u"Populus deltoides Bartr cv. 'LUX', I-69",
u'Populus deltoides Bartr, Dvina', u'Populus deltoides Bartr, Lena',
u"Populus deltoides Bartr. cv. 'Havard', NL-80351",
u"Populus deltoides Bartr. cv. 'Imperial'",
u"Populus deltoides Bartr. cv. 'LUX' x (Populus x euramericana, Guineir 'I-45/5
u"Populus deltoides Bartr. cv. 'LUX' x (Populus x euramericana, Guineir 'I-45/5
u"Populus deltoides Bartr. cv. 'LUX' x (Populus x euramericana, Guineir 'I-45/5
u"Populus deltoides Bartr. cv. 'LUX' x Populus deltoides Bartr. cv. 'Harvard',
u"Populus deltoides Bartr. cv. 'LUX' x Populus deltoides Bartr. cv. 'Havard'",
u"Populus deltoides Bartr. cv. 'LUX' x Populus deltoides Bartr. cv. 'Havard', N
u"Populus deltoides cl. '55/65' x Populus deltoides cl. '2KEN8' x (Populus nigr
u'Populus deltoides clone D121', u"Populus deltoides cv. 'LUX' ",
u'Populus deltoides cv. I-69/55', u'Populus deltoides cv.35',
u"Populus deltoides x Populus nigra 'Robusta', DN-17; Populus deltoides x Popul
u"Populus deltoides x Populus nigra cv. 'Vereecken'",
u'Populus deltoides x Populus nigra hybrid 145/51',
u'Populus deltoides x Populus nigra, Gaver',
u'Populus deltoides x Populus nigra, Gibecq',
u'Populus deltoides x Populus nigra, Primo',
u'Populus deltoides x Populus nigra, cv. Robusta',
u'Populus deltoides x Populus trichocarpa, IBW1',
u'Populus deltoides x Populus trichocarpa, IBW2',
u'Populus deltoides x Populus trichocarpa, IBW3',
u'Populus deltoides, I-69', u'Populus deltoides, Lux',
u"Populus euramericana Guinier cv. 'San Martino' x Populus deltoides Bartr cv.
u"Populus euramericana Guinier cv. 'San Martino' x Populus deltoides Bartr cv.
u"Populus euramevicana 'I-214'", u'Populus eurasia x yunn.',
u'Populus hybrid, D-01 (P trichocarpa x P. nigra or P.trichocarpa x P. angusti
u'Populus maximowiczii Henry x trichocarpa, OP42',
u'Populus maximowiczii x Populus berolinensis Oxford, P-494',
u'Populus maximowiczii x Populus nigra, AF10',
u'Populus maximowiczii x Populus nigra, Japan 105',
u'Populus maximowiczii x Populus nigra, NM5',
u'Populus maximowiczii x Populus nigra, NM6',
u'Populus maximowiczii x Populus trichocarpa (OP42)',
u'Populus maximowiczii x Populus trichocarpa, NE42',
u'Populus nigra',
u'Populus nigra x Populus maximowiczii Maxfunf, J-105',
```

```
u'Populus nigra x Populus maximowiczii Maxvier, J-104',
u'Populus nigra, Wolterson', u'Populus russkii',
u"Populus simonii Carr. x Populus pyramidalis Bge. cv. 'Chifengensis 34'",
u'Populus tremula x Populus tremula, Br11 x W52',
u'Populus tremula x Populus tremula, GrDb1 x W52',
u'Populus tremula x Populus tremula, W51 x W52',
u'Populus tremula x Populus tremula, W95 x W52',
u'Populus tremula x Populus tremuloides, Br11 x T44-60',
u'Populus tremula x Populus tremuloides, Br11 x Tur.141',
u'Populus tremula x Populus tremuloides, GrDb1 x T44-60',
u'Populus tremula x Populus tremuloides, GrDb1 x Tur.141',
u'Populus tremula x Populus tremuloides, W51 x T44-60',
u'Populus tremula x Populus tremuloides, W51 x Tur.141',
u'Populus tremula x Populus tremuloides, W95 x T44-60',
u'Populus tremula x Populus tremuloides, W95 x Tur.141',
u'Populus tremula x tremuloides, Ekebo',
u'Populus tremuloides x Populus tremula, Ihl3 x W52',
u'Populus tremuloides x Populus tremula, Ihl3 x W66',
u'Populus tremuloides x Populus tremuloides, Ihl3 x Tur. 141',
u'Populus trichocarpa Hook',
u'Populus trichocarpa Torr. & Gray x Populus deltoides Marsh.',
u'Populus trichocarpa Torr. & Gray, black cottonwood',
u'Populus trichocarpa Torr. and Gray',
u"Populus trichocarpa Torr. and Gray, clone 'Fritzi Pauley'",
u'Populus trichocarpa x Populus balsamifera, Balsam Spire',
u'Populus trichocarpa x Populus deltoides, Beaupre',
u'Populus trichocarpa x Populus deltoides, Boelare',
u'Populus trichocarpa x Populus deltoides, Hazendans',
u'Populus trichocarpa x Populus deltoides, Hoogvorst',
u'Populus trichocarpa x Populus deltoides, Hybrid 11',
u'Populus trichocarpa x Populus deltoides, Hybrid 5',
u'Populus trichocarpa x Populus deltoides, Hybrid 8',
u'Populus trichocarpa x Populus deltoides, Raspalje',
u'Populus trichocarpa x Populus deltoides, Robusta',
u'Populus trichocarpa x Populus deltoides, Unal',
u'Populus trichocarpa x Populus koreana (cf. Populus trichocarpa x Populus del
u'Populus trichocarpa x Populus trichocarpa, Trichobel',
u'Populus trichocarpa x deltoides (Beaupre)',
u'Populus trichocarpa x deltoides, Beaupre',
u'Populus trichocarpa x deltoides, Boelare',
u'Populus trichocarpa, Columbia River',
u'Populus trichocarpa, Fritzi Pauley',
u'Populus tristis, clone DN-34', u'Populus tristis, clone NC-5260',
u'Populus tristis, clone NC-5377', u'Populus tristis, clone NE-299',
u'Populus tristis, clone NE-386', u'Populus tristis, clone NE-41',
u'Populus x canadensis Monch, Bellini',
u'Populus x canadensis Monch, Luisa Avanzo',
u'Populus x canadensis Monch, Neva',
```

u'Populus x canadensis, 83.148.041', u'Populus x canadensis, AF2',
u'Populus x canadensis, I-124', u'Populus x canadensis, Sirio',
u"Populus x euramericana (Dode) Guineir cl. 'N2136'",
u"Populus x euramericana (Dode) Guinier cv. 'San Martino', I-72",
u"Populus x euramericana (Dode) Guneir cv. 'San Martino'",
u'Populus x euramericana I-214',
u'Populus x euramericana cl "Neva"',
u"Populus x euramericana cl. 'N3014'",
u"Populus x euramericana cl. 'N3016'",
u"Populus x euramericana cv. '74/76'",
u'Populus x euramericana, I-72',
u"Populus x xiaozhuanica W. Y. Hsu et Liang cv. 'Zhaoling-6'",
u'Purple, NG2', u'S. matsudana x ?, Drago',
u'S. matsudana x ?, S76-008', u'S. matsudana x ?, S768-003',
u'S. matsudana x ?,Levante',
u'Saccharum arundinaceum (Retz.) Jesw. cv. IK76-110',
u'Saccharum hybrid, L 79-1002', u'Saccharum officinarum L.',
u'Saccharum spp. cv. "L79-1002"', u'Saccharum spp. cv. CP89-2143',
u'Saccharum spp. cv. L79-1002', u'Salix Jiangsuensis Cl. 172',
u'Salix Jiangsuensis Cl. 549', u'Salix Jiangsuensis Cl. 795',
u'Salix alba', u'Salix alba L. and Salix matsudana Koidz.',
u'Salix alba S-64-004 x Salix alba SI62-016, 91.072.002',
u'Salix alba SE64-024 x Salix alba S-61-019, 91.089.024',
u'Salix alba SE66-023 x Salix alba S-61-002, 93.076.023',
u'Salix alba, SA2', u'Salix alba, SI68-015',
u'Salix alberti x Salix x leucopithecia, SE03-009',
u'Salix babylonica x Salix humboldtiana, SE64-010',
u'Salix burjactica Nasarow x Salix dasyclados Wimm., Gudrun',
u'Salix burjactica SW 901321 x Salix viminalis SW 881031, Doris',
u'Salix chelophila, Wuliu',
u'Salix dasyclados / Salix burjactica, Loden',
u'Salix dasyclados Helga x Salix dasyclados LV Rod, Gudrun',
u'Salix dasyclados Wimm. clone 81090',
u'Salix dasyclados x Salix dasyclados, Loden',
u'Salix dasyclados, ASV1', u'Salix dasyclados, Loden',
u'Salix dasyclados, SV1', u'Salix discolor Muhl.',
u'Salix discolor, S365', u'Salix erio 16 x Salix erio 24, S185',
u'Salix erio 16 x Salix erio 24, S546',
u'Salix erio 16 x Salix erio 24, S557',
u'Salix erio 16 x Salix erio 276, S25',
u'Salix erio 16 x Salix erio 307, S19',
u'Salix erio 19 x Salix erio 23, S652',
u'Salix erio 28 x Salix erio 24, S566',
u'Salix erio 28 x Salix erio 24, S646',
u'Salix erio 39 x Salix interior 42, S625',
u'Salix erio 39 x Salix petiolaris 47, S599',
u'Salix erio 62 x Salix erio 276, S301',
u'Salix eriocephala (erio), S287',

```
u'Salix eriocephala x Salix interior, S625',
u'Salix eriocephala, AS287', u'Salix eriocephala, AS625',
u'Salix eriocephala, S25', u'Salix eriocephala, S546',
u'Salix fragilis', u'Salix fragilis, SI72-001', u'Salix integra',
u'Salix interior x Salix eriocephala, S301',
u'Salix jessoensis SE63-016 x O.p., 90.090.003',
u'Salix jessoensis SE63-016 x O.p., 90.090.021',
u'Salix jessoensis SE63-016 x O.p., 90.090.023',
u'Salix jessoensis, SE63-016',
u'Salix koriyanagi x Salix purpurea, Allegany',
u'Salix koriyanagi x Salix purpurea, Onondaga', u'Salix matsudana',
u'Salix matsudana J-172',
u'Salix matsudana SE64-012 x O.p., 90.091.001',
u'Salix matsudana SE64-012 x O.p., 90.091.003',
u'Salix matsudana SE64-012 x O.p., 90.091.021',
u'Salix matsudana SE64-012 x O.p., DRAGO',
u'Salix matsudana SE64-012 x O.p., LEVANTE',
u'Salix matsudana SE64-012 x O.p., S76-003',
u'Salix matsudana SE69-002 x O.p., 90.092.022',
u'Salix matsudana SE69-002 x O.p., 90.092.023',
u'Salix matsudana SE69-002 x O.p., 90.092.024',
u"Salix matsudana x (Salix matsudana x Salix alba) 'V374', 'V461' and Salix al
u'Salix matsudana x Salix alba, AAUSC',
u'Salix matsudana x Salix alba, AAUSL', u'Salix matsudana x alba',
u'Salix matsudana, SE64-012', u'Salix miyabeana,  SX67',
u'Salix miyabeana, Canastota', u'Salix miyabeana, SX61',
u'Salix miyabeana, SX64', u'Salix miyabeana, SX67',
u'Salix miyabeana, Sherburne',
u'Salix purpurea x Salix miyabeana, Millbrook',
u'Salix purpurea x Salix miyabeana, Oneida',
u'Salix purpurea, 94001', u'Salix purpurea, 94003',
u'Salix purpurea, 94004', u'Salix purpurea, 94005',
u'Salix purpurea, 94006', u'Salix purpurea, 94009',
u'Salix purpurea, 94012', u'Salix purpurea, 94013',
u'Salix purpurea, 94014', u'Salix purpurea, 94015',
u'Salix purpurea, AFC189', u'Salix purpurea, AFC190',
u'Salix purpurea, Fish Creek', u'Salix purpurea, Pur12',
u'Salix purpurea, Pur34',
u'Salix redheriana x Salix dasyclados 77056, Endurance',
u'Salix sachalinensis, SX61',
u'Salix schwerinii E. Wolf x viminalis L. "Tora"',
u'Salix schwerinii E. Wolf. x Salix viminalis L., Bjorn',
u'Salix schwerinii Eucalyptus Wolf. x Salix viminalis L., Bjorn',
u'Salix schwerinii Hilliers x Bjorn, Discovery',
u'Salix schwerinii Hilliers x Jorr, Endeavour',
u'Salix schwerinii L79069 x Salix viminalis Orm, Tora',
u'Salix schwerinii x Salix viminalis, Tora', u'Salix spp., Tora',
u'Salix triandra SW911066 x Salix viminalis Jorr, Inger',
```

```
u'Salix viminalis', u'Salix viminalis Astrid x Bjorn, Asgerd',
u'Salix viminalis Astrid x Salix viminalis, Beagle',
u'Salix viminalis Bowles Hybrid x Bjorn (Salix schwerinii L79069 x Salix vimina
u'Salix viminalis Bowles Hybrid x Salix burjactica Korso, Ashton Stott',
u'Salix viminalis L.', u"Salix viminalis L. cv. Bowles 'hybrid'",
u'Salix viminalis L., Bowles Hybrid',
u'Salix viminalis L., clone 1047',
u'Salix viminalis L., clone 1054',
u'Salix viminalis L., clone 78021',
u'Salix viminalis L., clone 78083',
u'Salix viminalis Pavainen x Bjorn, Quest',
u'Salix viminalis cv. Jorunn',
u'Salix viminalis x Salix miyabeana, Fabius',
u'Salix viminalis x Salix miyabeana, Otisco',
u'Salix viminalis x Salix miyabeana, Owasco',
u'Salix viminalis x Salix miyabeana, Truxton',
u'Salix viminalis x Salix miyabeana, Tully Champion',
u'Salix viminalis x Salix schwerinii, Tora',
u'Salix viminalis x Salix viminalis, Jorr',
u'Salix viminalis x Salix viminalis, Jorunn',
u'Salix viminalis, Jorr', u'Salix viminalis, Jorunn',
u'Salix viminalis, L78183', u'Salix viminalis, Rapp',
u'Salix viminalis, SVQ', u'Salix viminalis, Salix 21',
u'Salix viminalis, clone 77-683', u'Salix viminalis, clone 78112',
u'Salix viminalis, clone 78195', u'Salix x dasyclados, SV1',
u"Salix x jiangsuensis CL 'J-172'", u'Secale cereale L.',
u'Secale montanum L., Permontra', u'Sida hermaphrodita L. Rusby',
u'Sorghastrum nutans L. Nash., Tomahawk, Toma',
u'Sorghum bhalepense Pers.', u"Sorghum bicolor L. Moench 'H133'",
u'Sorghum bicolor Moench',
u"Sorghum bicolor x Salix sudanense cv 'Southern States'",
u'Sorghum bicolor, cv. Kimia', u'Sorghum sudanensis',
u'Spartina cynosuroides', u'Spartina pectinata L., CWNC, CWNC',
u'Spartina pectinata Link.',
u'Thinopyrum intermedium (Host) Barkw. & D.R. Dewey, Thinopyrum ponticum (Podp
u'Thinopyrum intermedium (Host), Barkw. & D.R. Dewey, Intermediate wheatgrass'
u'Timor provenance',
u'Tora x Salix miyabeana Shrubby Willow, Nimrod',
u'Tora x Salix viminalis Orm, Torhild',
u'Tora x Salix viminalis Ulv, Tordis',
u'Triarrhena sacchariflora (Maxim.) Nakai',
u'Tripsacum dactyloides L.', u'Triticosecale Wittmack',
u'Zea mays L.', u'clone 075, Salix dasyclados Wimm.',
u'clone 077, Salix schwerini', u'clone 081, Salix viminalis L.',
u'clone 082, Salix viminalis',
u'clone 15-029, Populus trichocarpa Torr. and Gray x Populus deltoides Marsh.'
u'clone 206, Salix dasyclados x purpurea',
u'clone 44-133, Populus trichocarpa Torr. and Gray x Populus deltoides Marsh.'
```

```
u'clone 50-197, Populus trichocarpa Torr. and Gray x Populus deltoides Marsh.'
u'clone 55-272, Populus trichocarpa Torr. and Gray x Populus deltoides Marsh.'
u'clone 57-276, Populus trichocarpa Torr. and Gray x Populus deltoides Marsh.'
u'clone 59-289, Populus trichocarpa Torr. and Gray x Populus deltoides Marsh.'
u'clones. "Max-4"',
u'h-aspen, crosses between Populus tremula, L and Pupulus tremuloides Michaux'
u'h-poplar OP 42, Populus maximowiczii, Henry and Populus deltoides, Marshall'
u'mean of 25 clones',
u'mean of Miscanthus x giganteus and Miscanthus sinensis',
u'mixed species', u'var. sanguinea',
u"x Triticosecale Wittmack 'Triskell' and 'Amarillo'",
u"x Triticosecale Wittmack 'Triskell', x Triticosecale Wittmack 'Triskell' 'Ama
```

### 3.3.3 Planting_date, Harvest_year, Harvest_date, Age, Rotation, Density

```
In [57]: print '-'*80
         print 'Planting_date, unique values:\n', np.sort(df1.Planting_date.unique())
         print '-'*80
         print 'Harvest_year, unique values:\n', np.sort(df1.Harvest_year.unique())
         print '-'*80
         print 'Harvest_date, unique values:\n', np.sort(df1.Harvest_date.unique())
         print '-'*80
         print 'Age, statistical description:\n', df1.Age.describe()
         print '-'*80
         print 'Rotation, statistical description:\n', df1.Rotation.describe()
         print '-'*80
         print 'Density, statistical description:\n', df1.Density.describe()
```

```
--------------------------------------------------------------------------------
Planting_date, unique values:
[1958 1963 1967 1969 1970 1971 1973 1976 1977 1979 1981 1982 1983 1984 1985
 1986 1987 1988 1989 1990 1991 1992 1993 1994 1995 1996 1997 1998 1999 2000
 2001 2002 2003 2004 2005 2006 2007 2008 2009 2010 2011 2012 nan 2013
 u'1988-1993' u'1992-1993' u'2000-10' u'2000-3' u'2001-12' u'2001-3'
 u'2003-8' u'2004-4' u'2005-2']
--------------------------------------------------------------------------------
Harvest_year, unique values:
[1968 1969 1971 1973 1975 1976 1977 1979 1980 1981 1982 1983 1984 1985 1986
 1987 1988 1989 1990 1991 1992 1993 1994 1995 1996 1997 1998 1999 2000 2001
 2002 2003 2004 2005 2006 2007 2008 2009 2010 2011 2012 2013 2014 2015 nan
 2016 u'1972-1973' u'1976/1977' u'1988-1990' u'1990-1992' u'1991-1992'
 u'1992-1993' u'1993-1997' u'1994, 1995' u'1994-1996' u'1995-2001'
 u'1997-2012' u'1999-2000' u'2002-2005' u'2003-2005' u'2007-2011'
 u'2008-2011' u'2010-2011']
--------------------------------------------------------------------------------
Harvest_date, unique values:
[nan u'Apr' u'Aug' u'Aug, Nov' u'Aug, after senescence' u'Aug-Sep'
 u'Autumn' u'Autumn/winter' u'Dec' u'Dec, after senescence' u'Dec-Mar'
```

```
 u'Feb' u'Feb-Mar' u'Feb/Mar' u'Jan' u'Jan-Apr' u'Jan-Feb' u'Jan-Mar'
 u'Jul' u'Jul, Oct' u'Jul, Sep' u'Jun' u'Jun, Aug' u'Jun, Nov' u'Jun-Dec'
 u'Jun-Nov' u'Mar' u'Mar, Nov in 2010; Jul in 2011' u'May' u'May-Nov'
 u'Nov' u'Nov, after senescence' u'Oct' u'Oct, after senescence' u'Oct-Nov'
 u'Sep' u'after leaf fall' u'end of growing season'
 u'late winter/early spring' u'winter']
--------------------------------------------------------------------------------
Age, statistical description:
count    1876.000000
mean        4.901125
std         5.076235
min         0.830000
25%         2.000000
50%         3.000000
75%         6.000000
max        64.000000
Name: Age, dtype: float64
--------------------------------------------------------------------------------
Rotation, statistical description:
count    800.000000
mean       3.427500
std        2.073673
min        1.000000
25%        2.000000
50%        3.000000
75%        4.000000
max       12.000000
Name: Rotation, dtype: float64
--------------------------------------------------------------------------------
Density, statistical description:
count    2.828000e+03
mean     3.422405e+05
std      1.261370e+06
min      9.400000e+01
25%      2.290000e+03
50%      1.000000e+04
75%      2.000000e+04
max      1.000000e+07
Name: Density, dtype: float64
```

### 3.4 Yield

**Yield, Unit, Error, Error_type, Yield_type, Yield_type_Index, Yield_estimation, Yield_origin**

### 3.4.1 Yield, Unit, Error, Error_type

```
In [58]: print '-'*80
         print 'Yield, statistical description:\n', df1.Yield.describe()
         print '-'*80
         print 'Unit, unique values:\n', np.sort(df1.Unit.unique())
         print '-'*80
         print 'Error, statistical description:\n', df1.Error.describe()
         print '-'*80
         print 'Error_type, unique values:\n', np.sort(df1.Error_type.unique())
```

```
--------------------------------------------------------------------------------
Yield, statistical description:
count    5088.000000
mean       10.427969
std         7.685920
min         0.021000
25%         5.220000
50%         8.587500
75%        13.500000
max        54.300000
Name: Yield, dtype: float64
--------------------------------------------------------------------------------
Unit, unique values:
[u'ton DM/ha/yr']
--------------------------------------------------------------------------------
Error, statistical description:
count    367.000000
mean       1.349807
std        0.855765
min        0.000000
25%        0.700000
50%        1.290000
75%        1.830926
max        4.700000
Name: Error, dtype: float64
--------------------------------------------------------------------------------
Error_type, unique values:
[nan u'SD' u'SE']
```

### 3.4.2 Yield_type, Yield_type_Index

```
In [59]: print '-'*80
         print 'Yield_type_Index, value count:\n', df1.Yield_type_Index.value_counts()
         print '-'*80
         print 'Yield_type, unique values:\n', np.sort(df1.Yield_type.unique())
```

```
--------------------------------------------------------------------------------
```

```
Yield_type_Index, value count:
aboveground                   4248
part of aboveground            748
aboveground and belowground     92
Name: Yield_type_Index, dtype: int64
--------------------------------------------------------------------------------
Yield_type, unique values:
[u'200 largest-diameter stand aboveground biomass growth'
 u'5y mean annual increment of stemwood' u'Bole and Branch'
 u'Bole plus branches' u'Leafless aboveground dry mass'
 u'Stool biomass production' u'Weight increment' u'aboveground'
 u'aboveground + belowground biomass' u'aboveground biomass'
 u'aboveground biomass increment' u'aboveground biomass production'
 u'aboveground biomass productivity'
 u'aboveground biomass productivity, stem+branch+leaf+bark'
 u'aboveground biomass yield' u'aboveground dry biomass'
 u'aboveground dry biomass growth'
 u'aboveground dry biomass, average of different years'
 u'aboveground dry mass increment' u'aboveground dry weight'
 u'aboveground production' u'aboveground shoot biomass yield'
 u'aboveground woody biomass' u'aboveground woody biomass production'
 u'aboveground woody dry biomass' u'aboveground, 5cm'
 u'aboveground, harvested biomass' u'annual increment of stemwood'
 u'annual increment, aboveground biomass'
 u'annual increment, stem + branches > 3cm'
 u'annual increments of stem wood' u'annual net increment'
 u'annual production' u'annual yield' u'annual yield, aboveground'
 u'average annual yield' u'biomass' u'biomass dry weight'
 u'biomass dry yield' u'biomass harvested' u'biomass production'
 u'biomass yield' u'biomass yield, aboveground' u'dry biomass of shoots'
 u'dry matter production, aboveground' u'dry matter yield'
 u'dry matter yield, aboveground bimoass'
 u'dry matter yield, standing crop biomass'
 u'dry yield of aboveground biomass'
 u'dry yield of woodgrass, aboveground leafless biomass' u'forage yield'
 u'harvest biomass' u'harvest yield' u'leafless biomass'
 u'maximum mean annual biomass increment'
 u'mean annual aboveground biomass increment' u'mean annual increment'
 u'mean annual increment at yr 7' u'mean annual increment of dry matter'
 u'mean annual increment of stem wood'
 u'mean annual increment, aboveground biomass'
 u'mean annual increment, harvested' u'mean bole increment'
 u'plant biomass yield' u'shoot biomass' u'shoot biomass yield'
 u'shoot growth' u'stand aboveground biomass growth' u'standing biomass'
 u'stem + branch with bark' u'stem and branch biomass'
 u'stem and branch woody yields' u'stem biomass' u'stem biomass growth'
 u'stem biomass increment' u'stem biomass production'
 u'stem biomass productivity' u'stem growth' u'stem weight' u'stem+braches'
```

```
 u'stems' u'stems and branches' u'total aboveground biomass'
 u'total aboveground biomass production' u'total aboveground dry weight'
 u'total aboveground woody biomass' u'total aboveground yield'
 u'total biomass' u'total biomass (leaves, twigs, branches, bark, stem)'
 u'total biomass adjusted for survival, root+shoot' u'total biomass yield'
 u'total live woody yield'
 u'total net primary production, abovegroud biomass'
 u'total plant biomass yield (stem+leaves)'
 u'total yield aboveground biomass' u'wood+bark+branches'
 u'woody biomass production, stem' u'woody dry matter' u'yield' u'yield '
 u'yield, aboveground' u'yield, stem and branches without leaves']
```

### 3.4.3 Yield_estimation, Yield_origin

```
In [60]: print '-'*80
         print 'Yield_estimation, unique values:\n', np.sort(df1.Yield_estimation.unique())
         print '-'*80
         print 'Yield_origin, unique values:\n', np.sort(df1.Yield_origin.unique())
```

```
--------------------------------------------------------------------------------
Yield_estimation, unique values:
[nan u'allometric equation'
 u'converted from stem volume using a basic density' u'dried, weighted'
 u'dried, weighted, allometric equation' u'weighted']
--------------------------------------------------------------------------------
Yield_origin, unique values:
[u'averaged by Age' u'averaged by Age, multiplied by wood density'
 u'directly reported' u'mean value of range' u'multiplied by Density'
 u'multiplied by Density, averaged by Age'
 u'multiplied by Density, averaged by Age, unit conversion'
 u'multiplied by Density, unit conversion' u'read from Fig. 1'
 u'read from Fig. 1, unit conversion' u'read from Fig. 2'
 u'read from Fig. 2, averaged by Age'
 u'read from Fig. 2, multiplied by Density' u'read from Fig. 3'
 u'read from Fig. 3, averaged by Age'
 u'read from Fig. 3, averaged by Age, unit conversion'
 u'read from Fig. 3, unit conversion' u'read from Fig. 4'
 u'read from Fig. 4, multiplied by Density, unit conversion'
 u'read from Fig. 5, unit conversion' u'read from Fig. 6, unit conversion'
 u'read from Fig. 7' u'reported-volume multiplied by reported-wood density'
 u'unit conversion']
```

## 3.5 Management

**Management, Irrigation, Irrigation_flag, Fertilization_flag, Fertilizing_frequency, Nitrogen, Phosphorus, Potassium, Magnesium, Boron, Calcium, Other_fertilization**

### 3.5.1 Management

```
In [61]: print '-'*80
         print 'Management, unique values:\n', np.sort(df1.Management.unique())
```

```
--------------------------------------------------------------------------------
Management, unique values:
[nan u'0.5 kg mixed fertilizer per plant' u'1 harvest occasion'
 u'1 kg compound fertilizer per plant' u'1 kg mix fertilizer per plant'
 u'1-cut' u'100% maximum evapotranspiration restoration'
 u'1000 kg/ha Enmag (6% N, 9% P, 9% K and 10 Mg)' u'120 mg/kg N'
 u'150 mg/kg N' u'15N applied in 1994' u'15N applied in 1995'
 u'15N applied in 1996' u'1800 mg/kg N' u'1st generation' u'1st rotation'
 u'2 harvest occasion' u'2-cut'
 u'25% maximum evapotranspiration restoration'
 u'250 g mixed fertilizer per plant' u'2:1 intercropped' u'2nd generation'
 u'2nd rotation' u'3 harvest occasion' u'3-cut' u'30 mg/kg N'
 u'300 kg/ha Enmag (20% P2O5, 11%K2O) and 450 Kg/ha sulphate of ammonia (21% N)'
 u'33 Mg/ha farmyard manure in 2009' u'3rd generation' u'3rd rotation'
 u'4-cut' u'40 Mg/ha farmyard manure annually'
 u'40 kg/ha N in establishment year'
 u'483 kg of N and P2O5 and K2O from 16-16-16 per hectare, and herbicides'
 u'4th rotation' u'50% maximum evapotranspiration restoration'
 u'50% pruning' u'60 mg/kg N' u'70% pruning' u'750 kg/ha fertilizer'
 u'90 mg/kg N' u'900 kg lime/ha'
 u'Aug 2001: 2.5 t/ha quicklime and 160 kg/ha Potassium; Mar 2003: Rhe-Ka-Phos (10% P2O5 + 21%
 u'Aug 2001: 2.5 t/ha quicklime and 160 kg/ha Potassium; Mar 2003: Rhe-Ka-Phos (10% P2O5 + 21%
 u'Aug 2001: 2.5 t/ha quicklime and 160 kg/ha Potassium; Mar 2003: Rhe-Ka-Phos (10% P2O5 + 21%
 u'Broadcast seeding, seeding rate 11.2kg/ha'
 u'Broadcast seeding, seeding rate 16.8 kg/ha'
 u'Broadcast seeding, seeding rate 5.6 kg/ha'
 u'C, Conventional harvest management'
 u'Callus culture (Micro II), irrigated first year' u'Calvin soil'
 u'Compound fertilizer at plantation' u'Coppice Structure'
 u'Drilled seeding, seeding rate 11.2kg/ha'
 u'Drilled seeding, seeding rate 16.8 kg/ha'
 u'Drilled seeding, seeding rate 5.6 kg/ha' u'Fungicides once a year'
 u'Insecticide, fungicide' u'Irrigated' u'Irrigated, no fertilisation'
 u'Irrigation (4.4 * rainfall)' u'K-pattern plantation' u'Klinesville soil'
 u'N fertility initiated in 2007' u'N fertility initiated in 2008'
 u'N in the first year after establishment'
 u'Nitrogen fertilizer and irrigation' u'No N ' u'No fertilizer'
 u'No irrigation and fertilization' u'One-Cut' u'P-pattern plantation'
 u'Regular slash load, 1S' u'Rhizome division' u'Slash burnt, SB'
 u'Slash removed, 0S' u'Two-cut' u'Vitro tillering (Micro I)'
 u'Vitro tillering (Micro I), irrigated first year' u'W-pattern plantation'
 u'X, lax harvest management systems'
 u'ammonium nitrate every 3 weeks to maintain 3.2% levels in new leaf tissue'
 u'at beginning, 300 kg/ha (16%N, 7%P and 13%K)' u'average fertilization'
```

```
u'average irrigation'
u'average of management treatments (harvest twice a year, harvest in fall, and harvest in win
u'average over 100 and 170 kg N ha-1 fertility levels' u'bore water'
u'composted poultry manure' u'control' u'coppiced'
u'damage class 1, undamaged' u'damage class 2'
u'damage class 3, heavily damaged, shoot die-back, recoverd by resprouting of axillary buds'
u'defoliated, N+W+' u'defoliated, N+W-' u'defoliated, N-W+'
u'defoliated, N-W-' u'double harvest' u'drip irrigation with nutrients'
u'dry treatment by reduced soil water recharge'
u'early harvest treatments' u'effluent irrigation' u'ferterlised'
u'fertilier, pesticide' u'fertilised'
u'fertilised, SF: regular slash, followed by a localised application of an N, P and Zn mixture
u'fertilization' u'fertilization and irrigation'
u'fertilizer "8.24.24" (450 kg/ha)' u'fertilizer applied'
u'first rotation' u'greater water retention' u'herbicides'
u'herbicides applied in 200 L/ha of water'
u'heterogeneous: palnted randomly 3 times with 40 days inbetween'
u'high fertility level' u'high-fertility' u'intercropped'
u'interim harvest' u'interim harvest, Liquid manure' u'irrigated'
u'irrigation' u'irrigation during establishment year'
u'irrigation, fertilization' u'irrigation, no fertilization'
u'late harvest treatments' u'lesser water retention'
u'lime-stabilized sludge' u'lime-stabilized sludge + mulch'
u'low fertility level' u'main harvest' u'main harvest, Liquid manure'
u'manure (50 t/ha), pest control' u'micro-propagated' u'no N fertility'
u'no defoliated, N+W+' u'no defoliated, N+W-' u'no defoliated, N-W+'
u'no defoliated, N-W-' u'no fertilization' u'no fertilizer'
u'no irrigated' u'no irrigation' u'no irrigation, fertilization'
u'no irrigation, no fertilization'
u'no irrigation/fertilization/pest control' u'no pruning'
u'no weed control' u'non-irrigated' u'only at plantation year'
u'pest control' u'planted' u'planted on low land' u'planted on slope land'
u'planted on terrace' u'plastic mulch' u'plot center site'
u'plot edge site' u'pruning' u'rainfed' u'rhizome propagated'
u'rostore-fertility' u'sand burial' u'second rotation' u'single harvest'
u'single structure' u'solecropped' u'solid fertilized' u'straw ashes'
u'sum of two harvests' u'thinned' u'topsoil disturbed, SD' u'unfertilised'
u'uniform: plant on the same day' u'unthinned' u'weed control'
u'woody ashes']
```

### 3.5.2 Irrigation, Irrigation_flag

```
In [62]: print '-'*80
         print 'Irrigation, unique values:\n', np.sort(df1.Irrigation.unique())
         print '-'*80
         print 'Irrigation_flag, value count:\n', df1.Irrigation_flag.value_counts()
```

```
--------------------------------------------------------------------------------
Irrigation, unique values:
[nan 0 50 60 80 100 110 120 128.33333333333334 133 135 166.5 189.8 204
 230.25 400 405 430 440 450 496 600 701 730 744 796 846 900 914 1045 1095
 1116 1554 1715 1800 1825 2190 2700 u'2-6 cm/week during growing season'
 u'25% of maximum evapotranspiration, water restoration in soil'
 u'60 mm in 2010; 50 mm in 2011, no in 2012'
 u'75% of maximum evapotranspiration, water restoration in soil'
 u'Capillary irrigation tubes' u'Drip irrigation' u'Non-irrigated'
 u'Sprinkler' u'drip irrigation; waste water, containing 25% nitrogen'
 u'every 3 months for the first 2 years and later twice a year'
 u'first year' u'high' u'in establishment year'
 u'irrigation after planting' u'irrrigation when necessary' u'low'
 u'medium' u'no' u'no ' u'once in Apr and once in May 1996'
 u'only first year' u'only in establishment year'
 u'only the beginning of second growing season in 1991'
 u'periodic irrigation for the 1st season Of 1999'
 u'soil moisture is maintained to field capacity with drip irrigation'
 u'sprinkler once or twice per year as an emergency intervention' u'water'
 u'watered after plantation'
 u'watered every second day with rainfall equilvalent of 1.5 mm daily+rainfall'
 u'yes']
--------------------------------------------------------------------------------
Irrigation_flag, value count:
yes    785
no     489
Name: Irrigation_flag, dtype: int64
```

### 3.5.3 Fertilization_flag, Fertilizing_frequency, Nitrogen, Phosphorus, Potassium, Magnesium, Boron, Calcium, Other_fertilization

```
In [63]: print '-'*80
         print 'Fertilization_flag, value count:\n', df1.Fertilization_flag.value_counts()
         print '-'*80
         print 'Fertilizing_frequency, value count:\n', \
               df1.Fertilizing_frequency.value_counts()
         print '-'*80
         print 'Nitrogen, statistical description:\n', df1.Nitrogen.describe()
         print '-'*80
         print 'Phosphorus, statistical description:\n', df1.Phosphorus.describe()
         print '-'*80
         print 'Potassium, statistical description:\n', df1.Potassium.describe()
         print '-'*80
         print 'Calcium, statistical description:\n', df1.Magnesium.describe()
         print '-'*80
         print 'Boron, statistical description:\n', df1.Boron.describe()
         print '-'*80
```

```
print 'Calcium, statistical description:\n', df1.Calcium.describe()
print '-'*80
print 'Other_fertilization, unique values:\n', \
      np.sort(df1.Other_fertilization.unique())
```

```
--------------------------------------------------------------------------------
Fertilization_flag, value count:
yes    3226
no      788
Name: Fertilization_flag, dtype: int64
--------------------------------------------------------------------------------
Fertilizing_frequency, value count:
annual                                                       2173
one-time                                                      840
total in 3 years                                               66
total in the 6-year rotation                                   34
total in 6 years                                               12
total in 2 years                                                8
total in first 3 years                                          3
total of two applications  at plantation and at 7 months        2
Name: Fertilizing_frequency, dtype: int64
--------------------------------------------------------------------------------
Nitrogen, statistical description:
count    2822.000000
mean      113.086285
std       117.005633
min         0.000000
25%        57.000000
50%        90.000000
75%       134.000000
max      1600.000000
Name: Nitrogen, dtype: float64
--------------------------------------------------------------------------------
Phosphorus, statistical description:
count    1951.000000
mean       79.661798
std        88.484833
min         0.000000
25%        28.000000
50%        45.000000
75%       100.000000
max       700.000000
Name: Phosphorus, dtype: float64
--------------------------------------------------------------------------------
Potassium, statistical description:
count    1730.000000
mean       93.935886
std        84.546430
```

```
min         0.000000
25%        45.000000
50%        90.000000
75%       110.000000
max       797.000000
Name: Potassium, dtype: float64
--------------------------------------------------------------------------------
Calcium, statistical description:
count    166.000000
mean      48.521869
std       53.309046
min        0.000000
25%       13.569750
50%       20.710000
75%       60.000000
max      187.566700
Name: Magnesium, dtype: float64
--------------------------------------------------------------------------------
Boron, statistical description:
count    84.000000
mean      2.784921
std       2.279965
min       0.000000
25%       0.170000
50%       2.400000
75%       5.000000
max       8.000000
Name: Boron, dtype: float64
--------------------------------------------------------------------------------
Calcium, statistical description:
count      99.000000
mean      556.713430
std       286.482476
min        62.000000
25%       300.000000
50%       620.000000
75%       643.140000
max      1074.000000
Name: Calcium, dtype: float64
--------------------------------------------------------------------------------
Other_fertilization, unique values:
[nan
 u'100 kg/ha Granusol 2GB5 micronutrient fertilizer (5% Mn, 5% N, 5% Mg, 5% Fe, 1.5% Cu, 0.5% [illegible]
 u'100 kg/ha Granusol 2GB5 micronutrient fertilizer (5% Mn, 5% N, 5% Mg, 5% Fe, 1.5% Cu, 0.5% [illegible]
 u'100 kg/ha Granusol 2GB5 micronutrient fertilizer (5% Mn, 5% N, 5% Mg, 5% Fe, 1.5% Cu, 0.5% [illegible]
 u'11 kg/ha S, ' u'54 kg Cl; 55 kg S per ha'
 u'6 g/plant Fe; 3 g/plant Zn; 1 g/plant Mn' u'90 kg/ha total sulfur'
 u'Zn 5 kg/ha'
```

```
u'fertility level 1: 0-0.5-0.5 times of recommended N-P-K rate'
u'fertility level 1: 0.5-0.5-0.5 times of recommended N-P-K rate'
u'fertility level 1: 1-1-1 times of recommended N-P-K rate'
u'fertility level 2'
u'fertility level 2: 0-1-1 times of recommended N-P-K rate'
u'fertility level 2: 1-1-1 times of recommended N-P-K rate'
u'fertility level 3'
u'fertility level 3: 0-2-2 times of recommended N-P-K rate'
u'fertility level 3: 1-2-2 times of recommended N-P-K rate'
u'fertility level 3: 2-1-1 times of recommended N-P-K rate']
```
